# Current Therapeutic Cannabis Controversies and Clinical Trial Design Issues

**DOI:** 10.3389/fphar.2016.00309

**Published:** 2016-09-14

**Authors:** Ethan B. Russo

**Affiliations:** PHYTECSLos Angeles, CA, USA

**Keywords:** cannabis, clinical trials, drug abuse liability, cognition, driving, vaporization, placebo, pesticides

## Abstract

This overview covers a wide range of cannabis topics, initially examining issues in dispensaries and self-administration, plus regulatory requirements for production of cannabis-based medicines, particularly the Food and Drug Administration “*Botanical Guidance.*” The remainder pertains to various cannabis controversies that certainly require closer examination if the scientific, consumer, and governmental stakeholders are ever to reach consensus on safety issues, specifically: whether botanical cannabis displays herbal synergy of its components, pharmacokinetics of cannabis and dose titration, whether cannabis medicines produce cyclo-oxygenase inhibition, cannabis-drug interactions, and cytochrome P450 issues, whether cannabis randomized clinical trials are properly blinded, combatting the placebo effect in those trials via new approaches, the drug abuse liability (DAL) of cannabis-based medicines and their regulatory scheduling, their effects on cognitive function and psychiatric sequelae, immunological effects, cannabis and driving safety, youth usage, issues related to cannabis smoking and vaporization, cannabis concentrates and vape-pens, and laboratory analysis for contamination with bacteria and heavy metals. Finally, the issue of pesticide usage on cannabis crops is addressed. New and disturbing data on pesticide residues in legal cannabis products in Washington State are presented with the observation of an 84.6% contamination rate including potentially neurotoxic and carcinogenic agents. With ongoing developments in legalization of cannabis in medical and recreational settings, numerous scientific, safety, and public health issues remain.

## Introduction

Is there a pathway that will lead to the return of cannabis to mainstream medicine? The answer is clear, inasmuch as it has already commenced. It follows the same time-honored process that any pharmaceutical must attain to receive regulatory approval: proof of biochemical uniformity and stability along with safety and efficacy as proven by randomized clinical trials (RCT).

A prescription cannabis product must be standardized, consistent and display a quality equal to any New Chemical Entity that has passed muster as a pharmaceutical (Russo, 2006a; Russo et al., 2015). It must also possess a practical and suitable delivery system that minimizes patient risk, including intoxication, other aspects of drug abuse liability (DAL) or serious adverse events, such as pulmonary sequelae. An additional requirement is a supply chain that ensures security that it is being distributed to its intended target patients.

The status quo of cannabis medicine for most patients still involves the black market with its myriad risks and inherent lack of quality control. In the future, the desirable alternative would be a safe and effective evidence-based pharmaceutical solution that physicians prescribe with confidence, that pharmacists endorse and supply, and that government health services and third party payers will cover.

Garden variety cannabis as bought on the street cannot meet these criteria nor gain regulatory approval in most nations of the world. The biochemical variability of one chemovar to another is a primary challenge, while unregulated material may harbor pesticide residues, molds, bacteria, or heavy metals that endanger public health. The most common delivery system, smoking, imposes similar risks: chronic cough, phlegm production, bronchitis, and inhalation of pyrolytic by-products (Tashkin, 2013). Cannabis inhalation, whether by smoking or vaporizer produces a rapid peak in serum and brain concentrations that maximizes intoxication and possible reinforcement that are risk factors for DAL (Schoedel et al., 2011). Other routes of administration, e.g., transdermal patches and rectal suppositories have not yet demonstrated practicality in later stage clinical trials (Huestis, 2007).

A cannabis delivery system with reliable intermediate onset, allows dose titration without pulmonary dangers, relieves symptoms, and is biochemically uniform and defined would engender confidence of all parties. The first preparation to fulfill these criteria, currently in 27 nations around the globe, is nabiximols (US Adopted name, also known as Sativex®), an oromucosal spray produced from whole cannabis extracts whose effects begin in 15–40 min, allowing a therapeutic window for control of symptoms without intoxication. In reality, patients are not seeking altered states from their medicine, but rather relief of pain or other complaints. Other cannabis-based medicines that follow will necessarily be required to meet similar benchmarks.

It is the widespread belief of most scientists and many legal scholars that the only viable solution to the issues of medicinal cannabis is a pharmaceutical approach. Only in this way are regulatory standards fulfilled, and patient desires satisfied in safety. Such an approach can remove the current clandestine atmosphere surrounding cannabis and promote the kind of open and mutual therapeutic doctor-patient relationship, while maintaining legal status. Political efforts at legalization in certain American states and various countries will necessitate wrestling with issues of pharmaceutical cannabis preparations as compared to allowable claims in a less regulated market for herbal preparations.

While cannabis is usually thought of in relation to THC content, and its agonistic effects at the CB_1_ receptor, it requires emphasis that humans express individual endocannabinoid tone, that may be deficient under certain conditions, as has been suggested in migraine, fibromyalgia, and irritable bowel syndrome (Russo, 2004, 2016a), or alternatively may be excessive in obesity and metabolic syndrome (Kunos, 2007). Beyond THC, other cannabis components such as cannabidiol and tetrahydrocannabivarin act as neutral antagonists at CB_1_ (McPartland et al., 2015) that along with dietary manipulations with prebiotics and probiotics portend to provide potential treatment interventions for the latter (Russo, 2016b).

## Controversies in cannabis dispensaries, coffee shops, and self-administration

The singular controversy in this category is quality control. Short of a biochemical analysis by a certified laboratory employing verified phytocannabinoids standards, the cannabis consumer can have no real idea of the composition or consistency of the product that they purchase. “Strain names” are eminently malleable, and are as simple to alter as writing a new label to change one to match the most desirable chemovar *du jour.* The wary consumer should also be alert to the possibility of coliform and heavy metal contamination, areas that are infrequently tested on the black market, and are unlikely to have been completed on every batch (*vide infra).* This type of laboratory analysis is not simple; one should keep in mind that the lipophilic nature of cannabinoids was responsible for a 150-year gap between the identification of morphine in opium to that of tetrahydrocannabinol in cannabis (Gaoni and Mechoulam, 1964). The problem is compounded by the facts that technically almost all laboratories pursuing phytocannabinoid analyses in the USA are doing so illegally, most without benefit of a Schedule I license from the Drug Enforcement Administration (DEA). Additionally, many phytocannabinoid standards available commercially are reportedly suspect. There is additional difficulty in attempting to analyze cannabis confections, as the assays require ascertainment of cannabinoid content from a complex food matrix (Vandrey et al., 2015).

Legal state requirements on cannabis analysis, and packaging vary wildly from one jurisdiction to another, and are often rudimentary. The author believes that full cannabinoid and terpenoid profiles are necessary for proper decisions by consumers in both the medical and markets (Russo, 2011). The current vernacular nomenclature classifying cannabis chemovars as “sativa” or “indica” is scientifically indefensible (see Piomelli and Russo, 2016 for more detailed opinion). Rather, what is necessary is more complete data on a given chemovar's biochemistry and attributable pharmacology. One sophisticated approach to the issue combining those quantitative assays with additional subjective data on scent, taste and effects, dubbed PhytoFacts®, has recently been described (Giese et al., 2015).

## Can a botanical agent become a prescription medicine?

Plants have been the historical source of medicine for most of human history, and continue to account for the base material of an estimated 25% of modern pharmaceuticals (Tyler, 1993). While the herbal market is unfathomable to many consumers and their doctors in countries lacking suitable regulation of the practice, it is now a proven fact that prescription drugs of botanical origin can be approved as medicines in most nations. This requires standardization based on sound science (Russo, 2001). Botanical medicines can even meet rigorous requirement of the American FDA as has already occurred for one topical agent (Veregen®, an extract of green tea, *Camellia sinensis*), and one single component botanical isolate taken internally, Fulyzaq® (crofelemer, from *Croton lechleri*). These approvals were achieved by following a blueprint that was updated in August 2015 (Food and Drug Administration, 2015; accessed May 2016) *Guidance for Industry*: *Botanical Drug Products*:

http://www.fda.gov/downloads/drugs/guidancecompliance regulatoryinformation/guidances/ucm458484.pdf.

## Regulatory hurdles

Since botanical medicines represent combinations of components, particular attention is necessary to product composition, which may be defined through quality control methods including spectroscopic and chromatographic techniques, “chemical fingerprinting,” assays for certain markers such as specific phytocannabinoids or terpenoids, assays of bioactivity, controls on raw material and processes in manufacture, and process validation with batch analysis (Fan et al., 2006; Fischedick et al., 2010; Giri et al., 2010). If components of an extract are not already “Generally Recognized As Safe” (GRAS), clinical trials, safety-extension studies and rigorous quality control requirements all must be met. A botanical agent administered by a non-oral route, such as inhalation, requires additional pharmacology and toxicology documentation before initiation of RCTs.

The *Botanical Guidance* (Food and Drug Administration, 2015) defines that that a botanical raw material (BRM; i.e., crude herb) becomes a botanical drug substance (BDS) after it is processed through extraction, mixing, excipient addition, formulation and packaging in a manner that is defined, exacting and precise. The BDS must be examined for its pharmacokinetic (PK) and pharmacodynamic (PD) properties. Additional regulatory requirements in a given country may also include monitoring for contaminants due to heavy metals, pesticides, bacteria and fungi. The FDA dictates long-term animal toxicity studies in two species, and reproductive toxicity, genotoxicity, and carcinogenicity investigations. Subsequent human studies of effects on cardiac QTc, DAL, and trials in human subjects with renal or hepatic insufficiency are mandatory.

## The issue of herbal synergy

Whether cannabis components beyond THC contribute to its medicinal effects has been an issue of contention (Wachtel et al., 2002; Ilan et al., 2005). Certainly, some have advocated this concept of herbal synergy (McPartland and Russo, 2001; Williamson, 2001; Wilkinson et al., 2003; Russo, 2011), which is quite akin to combinatorial activity of endocannabinoids via “the entourage effect” of active and inactive metabolites (Ben-Shabat et al., 1998; Mechoulam and Ben-Shabat, 1999). Such synergy would be apparent under conditions in which the activity of a minor component complemented the major, diminished the adverse event profile, or otherwise contributed to a preparation's stability or efficacy. The data supporting CBD as a synergist to THC has been summarized in the past (Russo, 2006c), including its anti-anxiety benefits (Zuardi et al., 1982), it anti-psychotic effects (Zuardi et al., 1995; Leweke et al., 2005, 2012; Morgan and Curran, 2008), its inhibition of THC metabolism to the possibly more psychoactive 11-hydroxy-THC (Bornheim and Grillo, 1998), inhibition of glutamate excitotoxicity and ability to serve as an anti-oxidant (Hampson et al., 2000), its anti-inflammatory and immunomodulatory activity in its own right (Malfait et al., 2000; Costa et al., 2007). CBD and other phytocannabinoids and terpenoids (McPartland and Russo, 2001) act in synergy with THC (Whittle et al., 2001) through pharmacological potentiation, amelioration of adverse events, summation, pharmacokinetic, and metabolic modulation (Russo, 2011). More recent investigations have added to this foundation, demonstrating the ability of CBD to eliminate dose-response ceiling to pain in an animal model (Gallily et al., 2014), how the presence of cannabidiol allowed a statistically significant difference in 30% pain improvement in opioid-resistant cancer pain in humans as compared to placebo and a THC-rich cannabis extract (Johnson et al., 2010), and the contributions of cannabis terpenoids to herbal synergy in whole cannabis preparations (Russo, 2011; McPartland and Russo, 2014).

## Pharmacokinetics and cannabinoid dose titration

Among the challenges of formulating phytocannabinoids as medicines are their lipid solubility and slow, often erratic oral absorption. While it is sometimes suggested that cannabis smoking permits accurate dose titration due to its rapid onset, this approach likewise delivers high concentrations in a rapid manner (Huestis, 2007). While this is the *raison d'être* of recreational cannabis smoking, it is wholly unnecessary and, quite arguably, undesirable to therapeutic applications, especially treatment of chronic conditions. The risk of rapid intoxication could be a likely factor in a putative cannabis-based medicine being rejected for regulatory approval, as seems to have been the case for inhaled THC products in the USA (Hart et al., 2005). In contrast, outside of early dosage titration, most Sativex patients experience no psychoactive effect, and report subjective intoxication visual analog scales (VAS) of less than 10 out of 100, not statistically different to placebo. Such results refute contentions that euphoria or other psychoactive accompaniments are necessary for attainment of symptomatic relief (Robson, 2011; Wade, 2012). Only one study of herbal cannabis, employing 25 mg of material of 9.4% composition, taken in a single inhalation was able to demonstrate a “sweet spot” in which pain relief was attained without significant accompanying intoxication (Ware et al., 2007).

A marked reduction in cannabis-associated adverse events was observed in the Sativex development program when initial high doses (up to 128 sprays/day) and rapid titration in early studies were eschewed in favor of slower titration with lower allowances (up to 12 sprays/day; Novotna et al., 2011). Interestingly, these lower doses with fewer side effects have also been observed to correlate to higher efficacy of therapeutic benefit in cancer pain control (Portenoy et al., 2012). Similar endorsements of the efficacy of “low-dose” cannabis therapy, and the “Start low, and go slow” philosophy have been forthcoming from herbal cannabis usage (by Dustin Sulak, D.O. and other clinicians) in the community (Russo et al., 2015).

In general, 2.5 mg of THC is a threshold dose for patients without previous tolerance to cannabis, 5 mg is a moderate dose, 10 mg a large dose that may be problematic for naïve patients, while 15 mg or more at a time risks psychiatric adverse events (Grotenhermen, 2001; Sellers et al., 2013).

## Anti-inflammatory drugs and cyclo-oxygenase (COX) inhibition

The adverse event profile with attendant morbidity and mortality of non-steroidal anti-inflammatory drugs (NSAID) has been of great public health concern. In essence, COX-1 agents may precipitate gastric ulceration and hemorrhage, while COX-2 agents may increase myocardial infarction and cerebrovascular accident risks (Fitzgerald, 2004; Topol, 2004). Apparently the anti-inflammatory and analgesic effects of THC and CBD are achieved independently of this mechanism, as they produce no COX inhibition of either isozyme at physiologic doses (Stott et al., 2005). In fact, cannabinoids have demonstrated inhibitory effects on duodenal ulcer formation (Douthwaite, 1947), as has the cannabis sesquiterpenoid/CB_2_ agonist, beta-caryophyllene (Tambe et al., 1996).

## Cannabis and drug-drug interactions

Both THC and CBD are metabolized via the cytochrome P450 system. In *in vitro* and *in vivo* animal models undertaken by GW Pharmaceuticals (data on file), inhibitory effects were only seen at exposures significantly higher than the maximum dose-equivalents utilized in human clinical trials and no relevant induction of cytochrome P450 enzymes seen *in vitro* for human CYP1A2, CYP2C9, CYP2C19, and CYP3A4. Specific testing of drug-drug interaction in human subjects was undertaken with nabiximols (Stott et al., 2013), and no clinically relevant changes in levels of CBD and THC with concomitant administration of ketoconazole, rifampicin, or omeprazole. In extensive clinical application including complex drug regimens with opioids, tricyclic antidepressants, anticonvulsants, etc., no drug-drug interactions have been observed that would contraindicate or preclude use of nabiximols with any specific pharmaceutical, although additive sedative effects are always possible (reviewed in more detail Russo, 2006b). In nabiximols, these sedative influences are seemingly counteracted by CBD (Nicholson et al., 2004).

CBD can induce CYP2B isozymes, and in current observational and clinical trial usage of CBD (Epidiolex®) in intractable epilepsy in children at very high doses up to 25 mg/kg/d, some elevation of the N-desmethyl clobazam metabolite of the anticonvulsant clobazam have been observed, producing sedation that has responded to dose reduction of the latter drug without corresponding loss of anticonvulsant efficacy (Devinsky et al., 2016). Caution is also advisable with utilization of other benzodiazepines, and valproic acid.

## Blinding, placebo, and design issues in cannabis randomized clinical trials

The ability to produce adequate blinding in clinical trials of psychoactive drugs has been a difficult challenge in pharmaceutical development. It has long been known that the mere act of being in a clinical trial generates a certain degree of subjective improvement, probably as a result of the extra attention that the patient receives, and her/his desire to please the clinical staff and contribute to the welfare of similarly affected individuals. These placebo effects are aggravated when the RCT lacks objective measures (as in many pain and mood trials), when the drug in question is psychoactive (antidepressants et al.), or when the agent in question has a reputation as “miraculous” (as is certainly the case in public perception of cannabis). The latter problem certainly applies to the current excitement regarding cannabis treatment of epilepsy. In a recent observational study of cannabidiol-rich cannabis chemovar use (Press et al., 2015), the response rate for families moving to Colorado for treatment of their children was 47% vs. only 22% for those already state residents, and three times as great for those reporting >50% response!

The placebo effect has become increasingly problematic over time. In a landmark study with chilling implications (Tuttle et al., 2015), it was documented that in the interval 1990–2013, placebo responses in neuropathic pain studies increased significantly (*p* = 0.002), that while drug responses in pain in early studies decreased an average of 34.7% from baseline and were stable over time, producing 16.5% greater analgesia than placebo, or 1 point decrease in NRS. By 2013, treatment advantage over placebo decreased (*p* = 0.0003) with only an 8.9% decrease in pain over baseline. Additionally, placebo responses increased with sample size (*p* = 0.001), and study length (*p* = 0.05), with the worst differences by far in USA-based studies. The US FDA generally requires 12-week duration RCTs in Phases II-III in accordance with IMMPACT (Initiative on Methods, Measurement, and Pain Assessment in Clinical Trials) Guidelines (Dworkin et al., 2005), but this may be counterproductive to the success of the clinical trial!

This hurdle of demonstrating salient differences of cannabis-based medicines over placebo may have been adequately surpassed by nabiximols (Wright et al., 2012) in some instances. Nabiximols and its corresponding placebo are seemingly indistinguishable in appearance, color and taste due to the peppermint masking excipient. Approximately 50% of nabiximols trial participants in earlier studies had prior exposure to cannabis, but *post-hoc* analysis indicates no differences in efficacy or side effect profile in cannabis-experienced vs. -naïve subjects, and differential efficacy of Sativex to various MS symptoms. Specifically, no statistical differences were observed in the incidence of Euphoric Mood among patients with prior experience of cannabis vs. those who were cannabis-naïve (3% in each instance). No significant differences were noted for any other psychiatric adverse events. No differences were noted in the two groups with respect to efficacy of symptom control with Sativex (supporting the efficacy of blinding), in contrast to the CAMS study (Zajicek et al., 2003; Cannador extract vs. THC vs. placebo), wherein treatment allocation was correctly guessed by patients to a greater degree than expected. Virtually all clinical studies of inhaled cannabis have produced salient differences in psychoactive side effects in the *verum* group vs. placebo, with the exception of the single inhalation study (*vide supra;* Ware et al., 2007). Subsequent post-marketing studies of nabiximols have similarly supported the efficacy of blinding (Notcutt, 2013; Notcutt et al., 2012; Rekand, 2014).

Whereas, earlier studies of nabiximols in intractable spasticity in multiple sclerosis produced variable differences from placebo (Wade et al., 2004, 2010; Collin et al., 2010), more impressive results were attained through the institution of a randomized withdrawal study design (Novotna et al., 2011). In part A (*N* = 572), unbeknownst to patients, all received nabiximols titrated upwards to clinical response or side effects. After 1 month, all patient came in for re-evaluation at a “resupply visit,” at which time, only those who had demonstrated a 20% or greater improvement in numerical rating scales (NRS) of spasticity were continued in the study. These patients were then randomized to continue nabiximols at the prior number of sprays per day, or, the corresponding number of placebo sprays. After an additional 12 weeks of treatment, spasticity NRS scores favored nabiximols over placebo by a convincing margin (*P* = 0.0002). Over the full course of 16 weeks, mean spasticity improvement on nabiximols was 48%.

Unfortunately, application of the same randomized withdrawal study approach in a Phase III clinical trial of nabiximols in cancer pain unresponsive to optimized opioids failed to attain statistical significance (data on file, GW Pharmaceuticals).

Other strategies that may reduce placebo effect in cannabis RCTs would include limiting patient expectations: “This experimental drug may or may not help you,” treating patients in a neutral fashion, avoiding confounding ancillary benefits to trial participation, such as concomitant physical therapy; utilizing a delivery technique with slower pharmacokinetics (oral as compared to inhalation); and, utilization of cannabis-based preparations that limit THC-induced psychoactivity with cannabidiol and terpenoid buffers (Russo, 2011).

## Cannabis, drug abuse liability, and DEA scheduling

The issue of cannabis abuse and dependency remains quite controversial. A cannabis dependency syndrome has been posited (Budney et al., 2004), with an oft quoted figure of 9% of ever cannabis users becoming dependent at some point. In the USA, at least, these figures, which apply to “recreational” usage must be tempered by the fact that greater than 50% of patients admitted to substance abuse treatment programs are there by legal mandate as an alternative to prosecution or incarceration, and not always because of an actual addiction to cannabis. Other authorities opine that cannabis has a DAL lower than that of other legal and illicit agents (Hilts, 1994; Roques, 1998; Nutt et al., 2007). The relative addictive potential of a drug is ascertained by judging its attendant intoxication, reinforcement, tolerance, withdrawal and dependency. DAL requires additional determination of public health and legal data on its degree of abuse and diversion. The advent of the Internet has revolutionized promulgation of drug information to any inquisitive potential consumer.

Herbal cannabis is scheduled in international and national categories that generally designate it as addictive or dangerous, having severe abuse potential, and lacking any recognized medical utility. In contrast, Marinol®, a synthetic form of THC has been down-scheduled in countries where it is an approved pharmaceutical, to a category denoting a lesser potential for abuse or lower dependency risk, after documentation showed rare abuse or diversion to the black market (Calhoun et al., 1998). This precedent is one that could potentially be repeated with cannabis-based medicines once their safety and appropriate DAL risk is demonstrated.

Intoxication, as noted above, is rarely problematic in long-term usage of nabiximols. The reinforcement properties of a drug are mediated in part by the rapidity of its delivery (Samaha and Robinson, 2005). Nabiximols' onset of effects is 15–40 min, with peak activity in a few hours. This is considerably slower than most drugs of highest abuse potential. Smoked and injected drugs produce greater reinforcement with higher peak serum and brain levels (Samaha and Robinson, 2005). Nabiximols effect onset is intermediate between that of inhalation and that of oral administration. CBD modulates the psychoactivity of THC (Russo, 2006a; Morgan and Curran, 2008; Morgan et al., 2010a,b). Euphoric mood, while desirable to recreational cannabis users, would be a possible risk for abuse in medical users, but has been uncommonly observed with nabiximols (Wade et al., 2006, 2010; Robson, 2011). Given acutely, particularly in cannabis-naïve individuals, THC may produce typical reactions such as tachycardia, hypothermia, orthostatic hypotension, and dry mouth, but these are subject to rapid tachyphylaxis in chronic administration (Jones et al., 1976). The latter side effects have become less prominent with slow titration of nabiximols (Russo et al., 2008), and it has shown no dose tolerance with respect to its therapeutic benefits in MS or treating cancer pain after prolonged usage as long as several years (Wade et al., 2006; Notcutt et al., 2012; Serpell et al., 2013).

Information from nabiximols RCTs and safety-extension (SAFEX) studies does not indicate any particular evidence of reinforcement. Euphoric mood is reported in nabiximols clinical trials with under 2% incidence (GW Pharmaceuticals, 2011).

Tolerance is quickly established to various manifestations of cannabinoid intoxication: tachycardia, hypothermia, orthostatic hypotension, dry mouth, ocular injection, intraocular pressure decreases, etc. (Jones et al., 1976). In over 15,000 patient-years of experience, no dose tolerance to nabiximols has been observed, however, while therapeutic efficacy is maintained (Wade et al., 2006; Notcutt et al., 2012; Serpell et al., 2013) In SAFEX studies in MS and peripheral neuropathic pain, nabiximols doses have been steady or reduced after months or years of administration (Serpell et al., 2013; Koehler, 2014). Symptomatic pain control is maintained with slow continued improvement in non-progressive disorders.

The existence or severity of a cannabis withdrawal syndrome remains under debate (Smith, 2002; Budney et al., 2004). In contrast to reported withdrawal sequelae in recreational users (Solowij et al., 2002), 24 subjects with MS who volunteered to discontinue nabiximols after a year or more suffered no withdrawal symptoms meeting Budney criteria. While symptoms such as pain recurred after some 7–10 days without Sativex, symptom control was rapidly re-attained upon resumption (Wade et al., 2006). Similar safety was noted in a clinical randomized withdrawal trial in spasticity of MS (Notcutt et al., 2012), wherein 36 patients previously improved on Sativex showed no withdrawal symptoms of significance. Additionally, in a study of 136 MS patients taking Sativex for a mean of 334 days, sudden cessation, no withdrawal effects of associated adverse events were reported (Serpell et al., 2013).

While herbal cannabis has lowest overall dependency risk of commonly abused drugs (Hilts, 1994; Roques, 1998; Nutt et al., 2007), that of nabiximols is apparently lower yet, due to slower peak compared to smoking, low doses required for therapeutic efficacy, virtual absence of intoxication in normal usage, and freedom from withdrawal sequelae even after chronic administration. Finally, no known abuse or diversion incidents with nabiximols were reported (as of March 2013). Formal DAL studies with nabiximols have demonstrated its drug abuse potential to be equal to or less than that of Marinol, which is Schedule III in the USA (Schoedel et al., 2011).

The situation is particularly puzzling for cannabidiol, which seems to be a victim of guilt by association, in that it was placed in Schedule I of the US Controlled Substances Act of 1970 along with cannabis and THC as a placeholder (United States Commission on Marihuana and Drug Abuse, 1972), and has remained there ever since, in spite of meeting no criteria for intoxication, reinforcement, tolerance, withdrawal, or dependency.

## Cognitive issues and neuropsychiatric sequelae

Cognitive issues with cannabis usage have been reviewed in the past (Russo et al., 2002; Fride and Russo, 2006) with more recent data and analyses on an ongoing basis, many alleging permanent sequelae and even structural changes on imaging studies. A meta-analysis of these data is beyond the scope of this highlights article, but readers are referred to a current excellent systematic review (Walsh et al., 2016). Certain points must be emphasized. Such studies are usually retrospective analyses of recreational cannabis usage, most often at high doses on a chronic basis. Often there is little premorbid data on neuropsychological status, psychiatric status, socioeconomic milieu, concomitant substance use and other pertinent potential confounders. Additionally, one must seriously consider whether dangers inherent to chronic cannabis usage, particularly in high-risk situations (adolescents, pregnancy, etc.) are absolutely relevant to medical application of cannabis under optimal conditions with non-inhaled administration at low doses with optimized preparations designed to reduce adverse events to the greatest extent possible.

Impairment of short-term memory impairment has been observed after heavy chronic recreational cannabis usage but virtually disappears after a few weeks' abstinence (Pope et al., 2001). More recent studies are similarly encouraging with regards to the reversibility of any cannabis-associated cognitive sequelae. In brief, observed hippocampal volume changes after cannabis usage were apparently reversible with cannabidiol exposure or abstinence (Yucel et al., 2016). Lower grade-point averages associated with persistent cannabis usage in high school pupils lost statistical significance one controlling for concomitant alcohol and tobacco usage (Meier et al., 2015). Similarly, cannabis usage alone was not found responsible for IQ or performance differences in teens compared to cigarette smoking or other confounds (Mokrysz et al., 2016). A very wise analysis points out that with recent developments in cannabis policy, rational approaches would include adoption of regulated markets with controls upon age of access to usage, accurate information on dosing guidelines for consumers, and availability of more balanced material with respect to cannabidiol availability with lower relative THC content (Curran et al., 2016).

Studies of cognitive issues in purely medical cannabis users are comparatively quite few. Four patients in the Compassionate Use Investigational New Drug program employing NIDA cannabis for decades, and up to 10 grams/day of low potency cannabis were systematically examined while maintaining their customary daily intake (Russo et al., 2002). Mild difficulties were noted in attention and concentration and with acquisition of complex new verbal material, but without effect on higher level executive function. No pertinent attributable imaging findings were observed on MRI studies. One of the subjects continues to serve at an executive level in an investment firm.

Halstead-Reitan battery components have been analyzed from two nabiximols studies. In peripheral neuropathic pain with allodynia (Nurmikko et al., 2005), no differences were noted vs. placebo. In central neuropathic pain in MS (Rog et al., 2005), four of five subtests failed to demarcate from placebo. While the Selective Reminding Test did not deteriorate significantly on Sativex during the study, placebo patients improved unexpectedly (*p* = 0.009).

Subsequently, a study of 55 MS with spasticity patients on nabiximols vs. 52 on placebo revealed no significant differences in cognitive ability after 1 year of treatment as measured by the Paced Auditory Serial Addition Test (PASAT; Rekand, 2014).

Depression and anxiety have been possible sequelae of recreational cannabis (reviewed Fride and Russo, 2006), but slight improvements were noted with nabiximols in one study (Rog et al., 2005) on Hospital Anxiety and Depression Scales. No mood disorders have been evident in long-term usage. Cannabidiol appears very promising in this area, but no major clinical investigations have commenced.

Whether cannabis has an causative role in schizophrenia remains contentious (Muller-Vahl and Emrich, 2008), but is no clear etiological relationship is evident based on epidemiological data (Degenhardt et al., 2003; Macleod et al., 2006; Muller-Vahl and Emrich, 2008; Hickman et al., 2009; Macleod and Hickman, 2010). One might assume that a greater risk would be evident as a dose-related phenomenon. The low serum levels required for nabiximols therapy, coupled with the anti-psychotic properties of CBD (Zuardi and Guimaraes, 1997; Morgan and Curran, 2008; Leweke et al., 2012), would hopefully minimize such risks. Once more, the long-term adverse event profile of nabiximols would seem to indicate few symptoms of paranoia, thought disorder or similar changes (Wade et al., 2010; Robson, 2011; Notcutt et al., 2012).

One study indicating safety and efficacy of cannabidiol in psychosis equal to or greater than conventional treatment has been published (Leweke et al., 2012), while another Phase II study of CBD as Epidiolex with positive results vs. placebo has been reported online and in the press.

## Immune function

Deleterious effects of cannabinoids on immune function have been reported in laboratory animals at doses 50–100 times the psychoactive threshold in humans (Cabral, 2001). No changes in leukocyte, CD4 or CD8 cell counts were documented in the Compassionate Use IND subjects (Russo et al., 2002). MS patients in the CAMS study of Cannador showed no immune changes (Katona et al., 2005), nor were they reported with smoked cannabis in a short-term HIV trial (Abrams et al., 2003). Hematological parameters have been unaffected in all nabiximols studies.

## Driving safety

While it is scientifically accepted that alcohol impairs driving performance, and that blood ethanol levels may accurately assess inherent risks, similar correlations to cannabis usage produce no such clear profile.

Some retrospective studies of road crashes impute an etiological relationship to recreational cannabis usage, while others (Movig et al., 2004) have not supported a link, unless cannabis was used along with alcohol. In a comprehensive review (Hadorn, 2004), the overall impression was of a low risk for cannabis in such accidents, and one less pronounced than that associated with benzodiazepines and older antihistamine formulations (Verster and Volkerts, 2004). A recent conference report also supports these findings (Soderstrom et al., 2005).

The situation is even less clear with respect to driving and medicinal cannabis. For Marinol, the manufacturer indicates http://www.drugs.com/pro/marinol.html#s28 (accessed February 2013): “Patients receiving MARINOL® capsules should be specifically warned not to drive, operate machinery, or engage in any hazardous activity until it is established that they are able to tolerate the drug and to perform such tasks safely.”

The Sativex Consumer information in Canada http://www.bayer.ca/files/SATIVEX-PM-ENG-PT3-30MAR2012-149598.pdf (accessed February 2013) states:

Serious Warnings and Precautions.
THC, one of the principal active components of SATIVEX®, has numerous effects on the central nervous system such as changes in mood, decreased mental performance and memory and altered perceptions of reality. Symptoms such as fainting and interference in the physical ability to carry out complicated tasks have been seen in patients taking SATIVEX®. Therefore, you should not drive, operate machinery or engage in activities that require unimpaired judgment and coordination.
While taking SATIVEX® you should not drink alcohol or take other drugs which may have an effect on the central nervous system such as sedatives or hypnotics, without consulting your doctor, as these products have a further additive effect on some of the symptoms listed above.

The CBD content of nabiximols may modulate THC effects (Russo, 2011). In a Phase I trial of Sativex in normal subjects, CBD promoted alertness and eliminated residual THC effects the morning following bedtime dosing (Nicholson et al., 2004). Additionally, *post-hoc* analysis of safety-extension patients with MS taking Sativex for over 1 year indicate that in the 73% of 119 subjects completing a questionnaire, 59% noted an improvement in total disability, 63% improved in at least one activity, 20% reported a decreased need for equipment or assistance, 95% noted positive changes in General Life Benefits, and 12–32% of caretakers noted easier administration to demands of activities of daily living.

Recently, nabiximols has been specifically tested for its effects on driving in 33 female MS patients with moderate to severe spasticity before and after 4–6 weeks of drug exposure (Rekand, 2014). No significant differences were noted in Visual Pursuit, Cognitrone, Reaction, Adaptive Tachistoscopic Traffic Perception, or Overall scores, while Determination was significantly improved by treatment (*P* = 0.026). Similarly, a survey of 196 patients in the Spanish patient registry revealed complaints of decreased driving ability after treatment of only 2.4%.

An expert panel (Grotenhermen et al., 2005) performed a comprehensive analysis of the issue of cannabinoids and driving with recommendations of as roadside sobriety tests, as opposed to *per se* standards of inactive cannabinoid metabolites that indicate past usage without accurate assessment of a driver's actual contemporaneous ability. The panel did endorse the validity of measures of THC itself (Grotenhermen et al., 2005, p. 7):

- Based on the results of culpability studies and from meta-analyses of experimental studies, *per se* laws for DUIC (driving under the influence of cannabis) should specify a legal limit for THC in blood serum of 7–10 ng/mL as a reasonable choice for determining relative impairment by cannabis. This corresponds to THC concentration in whole blood- the parameter commonly used in U.S. jurisdictions- or 3.5–5 ng/mL.

Of note, no studies demonstrated relevant impact of cannabis on driving skills at plasma levels below 5 ng/ml of THC. The latter benchmark value was incorporated into the legalization statute approved by voters in Washington State in 2012, and Colorado and Montana adopted this standard, as well.

A timely report sponsored by the American Automobile association has re-examined the issue (Logan et al., 2016): https://www.aaafoundation.org/sites/default/files/EvaluationOf DriversInRelationToPerSeReport.pdf.

This study examined 602 drivers arrested for driving under the influence (DUI) who tested positively for THC but no other substances, as well as 349 people with no drugs in their system. All the people had complete records as to their performance on tests by a Drug Recognition Expert (DRE). Their examination comprised physiological standards and psychophysical tests, including the Standardized Field Sobriety Test (SFST) battery. Also considered were data from an additional 4799 drivers testing positive for cannabinoids for whom testing results were available. Key findings included the following:

1. DRE arrestees performed more poorly than drug-free controls on walk-and-turn, one-leg-stand, and finger-to-nose tests.
2. Physical signs including red, bloodshot and watery eyes, tremor of eyelids, lack of ocular convergence, and rebound pupillary dilation were significantly more frequent in people testing positive for cannabis.
3. Data were analyzed for salient differences above and below the 5 ng/mL standard, but this was significant solely for the finger-to-nose test, wherein more misses were counted in the group with higher serum levels.
4. Data were additionally analyzed to ascertain if a different value from 1 to 10 ng/mL produced consistency with SFST results, but none was identified.
5. Arrests for DUI included 70 percent with THC concentrations below 5 ng/mL. While 23% of those detained were positive solely for THC, the majority tested positive for other drugs with or without alcohol.

Based on these data, laws including *per se* standards for impaired drivers for cannabinoids should be re-examined and replaced with roadside tests of impairment, followed by blood or other corroboratory laboratory tests where a problem is suggested.

## What about the children?

The press frequently criticizes medicinal cannabis on the basis that acquiescence to its availability promotes usage by youth. To the contrary, analyses such as that undertaken by the US Government Accounting Office (GAO) reveal no increase in associated drug crimes or youth usage rates after passage of state laws allowing medicinal cannabis (U.S. General Accounting Office, 2002; O'Keefe and Earleywine, 2005). Subsequent studies have failed to show any systematic increases, and in fact, compelling epidemiological investigation suggests a decrease in opioid overdose mortality in states that have legalized medicinal cannabis usage (Bachhuber et al., 2014). Additional support for opioid sparing comes from a recent investigation and accompanying editorial revealing decreased analgesic prescriptions in Medicare Part D in states where cannabis was available for medical purposes (Bradford and Bradford, 2016; Dyer, 2016).

There is no doubt that such concerns regarding youth usage are well-intentioned, but once more, as in all of clinical medicine, the recommendation of drug therapy requires informed consent and a full consideration of costs and benefits. Two recent studies of cannabis use in pregnancy seems to provide relative reassurance of lack of data to support birth defects, significant intrauterine growth retardation, or cognitive sequelae (Gunn et al., 2016; Torres and Hart, 2016). Eventually, cannabis based medicines will become available for serious pediatric conditions, such as nausea and vomiting with in chemotherapy and supportive oncology (Abrahamov and Mechoulam, 1995), primary treatment of cancer (Foroughi et al., 2011), cystic fibrosis (Fride, 2002), and severe neurologic impairment (Gottschling, 2011), and these concerns will require ongoing consideration.

## Smoking and vaporizers (adapted and updated from russo and hohmann, 2013)

Available formal RCTs of smoked cannabis have all been Phase II studies of quite short duration, and would have little sway among regulators in most nations. The IMMPACT recommendations for clinical trials in neuropathic pain, as one example (Dworkin et al., 2005) suggest a 12-week course of study. The cumulative cannabis exposure in herbal cannabis studies undertaken in California totaled only 1106 patient-days, or 3 patient-years, as analyzed in Russo and Hohmann (2013). In comparison, total experience with Sativex including clinical trials, prescription monitoring and named-patient supplies exceeded 30,000 patient-years in 2014, with much lower rates of side effects (data on file, GW Pharmaceuticals; Russo, 2006a, 2008).

The studies of smoked or vaporized cannabis to date have been completed with cannabis-experienced patients in almost every instance, usually as a protocol requirement. Whether the results can be generalized to cannabis-naïve patients is open to serious question.

Careful epidemiological studies support that cannabis smoking induces chronic cough and bronchitis (Tashkin, 2005), but seemingly not emphysema (Tashkin et al., 1997) or aerodigestive cancers (Hashibe et al., 2006). Lester Grinspoon noted (Grinspoon and Bakalar, 1997, p. 250), “—the only well-confirmed deleterious physical effect of marihuana is harm to the pulmonary system.”

In Canada, mainstream and side stream smoke of cannabis vs. tobacco smoke were compared (Moir et al., 2008). Their cannabis sample's smoke yielded ammonia (NH_3_) at a rate of 720 μg per 775 mg cigarette, 20 times higher than that in tobacco smoke, possibly due to usage of synthetic nitrate fertilizers. Formaldehyde and acetaldehyde were generally less concentrated in cannabis smoke than in tobacco, but butyraldehyde titers were higher. Polycyclic aromatic hydrocarbons were qualitatively similar. Levels of NO, NO_x_, hydrogen cyanide and aromatic amines concentrations were 3–5 times higher in cannabis smoke, with potential mutagenic and carcinogenic effects. Possible genotoxicity has been posited to cannabis smoke due to acetaldehyde production and production of possible DNA-adducts (Singh et al., 2009).

**Figure 1:**
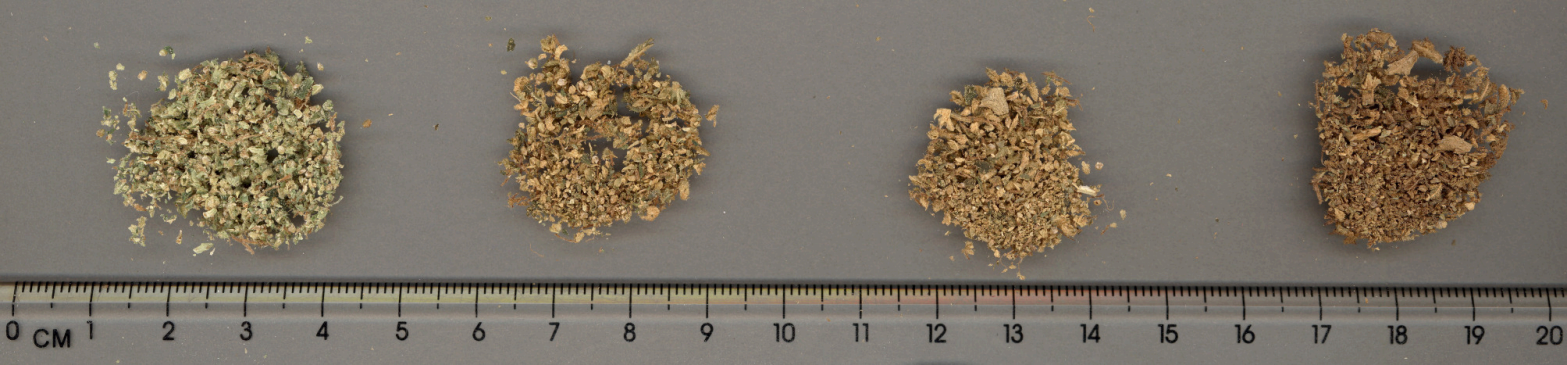
**Demonstration of the effects of vaporization of cannabis flower material at different temperatures employing the Volcano Digit Vaporizer (Left to Right): Unheated dried cannabis, Post-vaporization at 175°C, Post-vaporization at 195°C, Post-vaporization at 230°C**.

Vaporization of cannabis is designed to heat to a temperature that volatilizes THC and other components with the intent to minimize combustion by-products (Figure 1; For reviews please see Gieringer, 1996, 2001; Storz and Russo, 2003; Gieringer et al., 2004; Hazekamp et al., 2006; Bloor et al., 2008; Van der Kooy et al., 2008; Zuurman et al., 2008; Pomahacova et al., 2009).

The Volcano vaporizer was compared to smoking (Abrams et al., 2007) in 18 customary cannabis smokers, after 2 days of abstinence. NIDA 900 mg cannabis (1.7, 3.4, 3.4, and 6.8% THC) cigarettes were split in two, allowing one-half each to be smoked or vaporized sequentially in double-blind fashion. THC plasma concentrations were comparable or slightly higher after vaporization than smoking. Exhaled CO diminished very slightly in vapor, while it increased after smoking (*p* < 0.001). Visual analog scales of intoxication were almost identical and increased with higher potency cannabis. While it was claimed that since CO did not rise after vaporization, there would be “little or no exposure to gaseous combustion toxins” (p. 576), a quite remarkable assertion, given that PAHs and other products of combustion were not directly measured. It was also claimed that there were no reported adverse events. While twelve 12 experimenters preferred the Volcano, 2 liked smoking, and 2 had no preference as to technique. The authors labeled the vaporizer, “an acceptable system,” providing “a safer way to deliver THC—”(p. 576).

The Volcano Medic® with an upper temperature limit of 210°C. is a licensed medical device in the European Union, and in Canada since 2010 (No. 82405).

Subsequently, new innovations have appeared on the vaporization scene. The Syqe vaporizer from Israel is a hand held portable device designed to provide dose-metered single inhalations from ground herbal Bedrocan cannabis from the Netherlands (Eisenberg et al., 2014). This seemingly promising approach produced salient issues, nevertheless:

1. The observed maximum serum concentration of THC (Cmax) of 38 ng/ml is still in the psychoactive range, and would be an issue for regulators, as well as being possibly problematic for THC-naïve patients.
2. The selected patients with neuropathic pain were a biased population by virtue of already being cannabis patients.
3. Critics may point out that cannabis users will recognize the high, and confabulate that with reduced pain (whether that is true or not).
4. The study was unblinded, which would have no weight in the regulatory environment.
5. A 12-h abstention from the patients' customary therapeutic cannabis usage was employed in the trials, and is not long enough to eliminate residual effects on pain threshold from prior use. In most RCTs of clinical application of cannabis, a month of abstinence is mandated.
6. The pain relief was very brief (about 90 min), and use of this technology for a chronic pain patient would require very frequent application, thereby increasing relative risks of reinforcement and dependency. From a DAL standpoint, this would be unacceptable to most regulators.
7. The presence or absence of polyaromatic hydrocarbons was not assayed, and would more likely be lower employing a cannabis extract without extraneous plant material.
8. A 20% device failure was observed without additional explanation or qualification.

A recent study examined several newer vaporizer models (Lanz et al., 2016), all of which produced effective decarboxylation of the herbal Bedrocan cannabis base material. The highest cannabinoid recovery rates in the vapor were produced by the Arizer Solo®, 67.5–82.7%, but once more, no specific assays were performed for polyaromatic hydrocarbon residues.

As with smoked cannabis studies, clinical trials undertaken to date with vaporizers have been small pilot studies of maximum 5 days' duration, that will not likely portend regulatory acceptance under the *Botanical Guidance* (Food and Drug Administration, 2004). Neither is it probable that the side effect of smoked or vaporized cannabis would pass regulatory muster. Even if a perfect vaporizer were produced, standardization of the cannabis employed and quantification of delivered doses would be necessary for such approval, as well as total elimination of potential carcinogens and mutagens (Russo, 2006a). For better or worse, vaporizers have had little market penetration to date: an Internet survey noted that only 2.2% of cannabis users primarily utilized vaporization for cannabis consumption (Earleywine and Barnwell, 2007), but this situation may be in flux.

Cognitive effects evident in smoked cannabis studies call into question the reliability of blinding vs. placebo (*vide supra*). It is instructive to compare adverse event profiles for other drugs used for similar conditions: An analysis of medications employed for chronic polyneuropathy revealed that intolerable side-effects were not significantly different in cohorts receiving gabapentinoids, tricyclic anti-depressants, anticonvulsants, cannabinoids (including nabilone, Sativex) and topical agents (Toth and Au, 2008). None of the adverse events were serious.

## Cannabis concentrates: “dabs, wax, and shatter”

The illegality of cannabis of the last few decades has catalyzed selective breeding for ever more potent THC-predominant chemovars, and preparations that further concentrate that component. Whereas, techniques such as water hash or sieving of cannabis could produce *kif* or hashish of up to 60% THC (Clarke, 1998), chemical extractions with naphtha, petroleum ether, butane and other solvents can push these figures to 90% THC or more, with dangers of residual solvents (Romano and Hazekamp, 2013), fires and explosions in kitchen laboratories. A clean concentrate can be produced via supercritical CO_2_ extraction (Guy and Stott, 2005), but this requires technical equipment and expertise.

**Figure 2:**
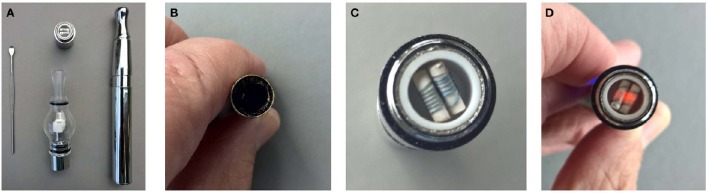
**Demonstration of a “vape-pen.” (A)** Vape-pen unit **(B)** “Wax” in unit **(C)** Unheated coil **(D)** Coil becomes red hot in seconds after actuation.

These cannabis concentrates sport various names: dabs, wax, shatter, and have spawned an entire new industry based on “vape pens” (Figure 2). The appellation may be a misnomer, however, as evidenced by the instant production of red hot elements in most devices. Their ramifications can be dramatic, as evidenced by increasing emergency visits for exploding lithium batteries in the units, or sequelae of their use in the form of panic reactions, toxic psychosis episodes, and orthostatic hypotension with resultant falls and accompanying injury. Even surveyed users acknowledge attendant greater tolerance and withdrawal effects (Loflin and Earleywine, 2014). The extreme viscosity of “wax” often necessitates the addition of propylene glycol and glycerol as propellants for the devices to function. Whereas, these substances are safe for oral usage in small amounts, recent research on their application in E-cigarettes containing nicotine reveals that when overheated, up to 2% of the mixture forms formaldehyde, a Group I carcinogen, producing a cancer risk estimated to be as much as 15 time that of chronic cigarette smoking (Jensen et al., 2015).

Many of these preparations are questionable with respect to their appropriateness to medicinal application. It is hoped that these by-products of prohibition with their advantage of clandestine transport of ever higher potency materials will become less attractive in a legal, regulated market.

## Cannabis contaminants: heavy metals, microbes and recent data on pesticides

Cannabis is a bio-accumulator (McPartland et al., 2000) that recruits heavy metals (lead, mercury, cadmium, and arsenic) from the soil into the plant biomass. While this is an advantage when rehabilitating contaminated soils with a hemp crop, it is a distinct liability if medical grade cannabis is grown in such media.

Similarly, foods and other materials to be ingested must be free of microbial contamination. Potential pathogenic bacteria may be introduced from the soil, fertilizer, or human handling with inadequate hygiene (i.e., handwashing). While clean culture without coliform bacterial infestation is certainly possible (Potter, 2009), some government-approved medicinal cannabis programs have utilized gamma-irradiation of cannabis for its sterilization. These raises some concerns of its own, while no residual radiation is reported in such instances, it is the case that no safety studies have been published to attest to the safety of the technique for a smoked or vaporized product. Additionally, gamma irradiation significantly reduced monoterpene content in orange juice and cilantro (coriander herb; Fan and Gates, 2001; Fan and Sokorai, 2002). A recent report in this journal examining the technique with cannabis has demonstrated “minor” quantitative changes in terpenoid content, if not overall profile (Hazekamp, 2016). The gamma irradiation did produce a 10% decrement in β-caryophyllene in one cannabis chemovar, and this certainly might affect the therapeutic effect of a medical product given that this sesquiterpenes component is a selective CB_2_ full agonist (Gertsch et al., 2008) with important anti-inflammatory and analgesic benefits (Russo, 2011, 2016b). Even without such changes, a certain segment of health-conscious consumers may choose to avoid such irradiated preparations on moral grounds, and deserve full disclosure in this regard.

Both the American Herbal Products Association (American Herbal Products Association, 2014) and American Herbal Pharmacopoeia (Upton et al., 2013) have developed guidelines for cultivation and production of medicinal cannabis. Contamination risk by bacteria and insects is greatest in the indoor environment that has predominated in the black market, is less in greenhouses, and less still in open air cultivation.

An informal survey in 2014 of California laboratories performing assays for residual pesticides on cannabis crops observed an incidence of only 1–2% (Backes, 2014). This author contacted other prominent California analytical laboratories that observed that 15–35% of samples submitted to them were positive, and that they had become hesitant to publicize their service or list agents for which they could assay, as they suspected that such information merely led unscrupulous growers to seek out possibly more toxic agents that were not included. A more formal published survey of contamination in California demonstrated qualitative presence of eight pesticides in 33% of samples (Raber et al., 2015).

Washington State legalized medicinal cannabis in 1998, where it remained largely unregulated until recently. Legalization of recreational cannabis was passed by ballot initiative in 2012, but despite panel recommendations, no testing for pesticide contamination was mandated. Subsequently, efforts are underway to unify the prior medical market with the legal one. No current method is available to certify organic cannabis culture, and there are no Environmental Protection Agency guidelines on acceptable pesticide levels for a smoked product. Prior informal testing in Washington was undertaken by a concerned medical purveyor yielded pesticide residues in 5–10% of tested cannabis inflorescence samples. An alarming recent study has demonstrated the passage of up to 70% of pesticides spiked into herbal cannabis into the captured smoke (Sullivan et al., 2013).

To more fully assess the current situation, 26 distinct cannabis samples were purchased (24 concentrates, 2 cannabis inflorescence) from legal stores in Washington and passed via witnessed chain of evidence to a state certified legal licensed laboratory (Trace Analytics, Spokane, WA; Russo, 2016c). Samples were homogenized, and extracted using a modified QuEChERS AOAC protocol. The supernatant was injected for LCMS-MS analysis. Detection was carried out using a Shimadzu LCMS-8050 triple quadrupole mass spectrometer with a Shimadzu Prominence HPLC. Approximately 200 analytes were measured with over 500 MRM transitions per run.

Out of the 26 Washington State samples, 22 tested positively for pesticides (84.6%). Many harbored multiple contaminants, attaining levels in the tens of thousands of parts per billion (ppb), exceeding the upper limit of quantification. These included 24 distinct pesticide agents of every class (Table 1; McPartland et al., 2000; Upton et al., 2013; Kegley et al., 2014): insecticides, miticides, fungicides, an insecticidal synergist and growth regulators, including organophosphates, organochlorides, carbamates, etc. One tested cannabis extract, a candidate for folding into the medical market in Washington, demonstrated lower levels of azoxystrobin, triflumizole, and piperonyl butoxide, with extreme levels of carbaryl, boscalid, bifenazate, pyraclostrobin, fenpyroximate, and myclobutanil, with documented associated toxicities as carcinogens, neurotoxins, cholinesterase inhibitors, developmental and reproductive toxins, and endocrine disruptors.

**Table 1:** **Pesticides encountered in 26 cannabis samples in Washington State, with structure, chemical class, and potential toxicities**.

**Pesticide**	**Structure**	**Class/Usage**	**Toxicity**
Azoxystrobin	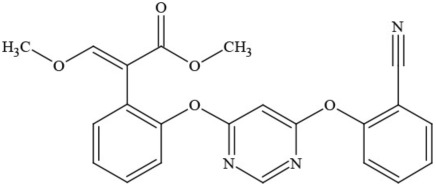	Fungicide	Questionable developmental/reproductive toxin and endocrine disruptor
Bifenazate	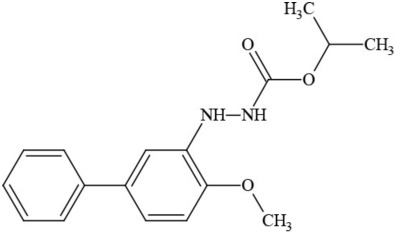	Miticide	Slight acute toxicity, potential ground water contaminant, questionable developmental/reproductive toxin and endocrine disruptor
Boscalid	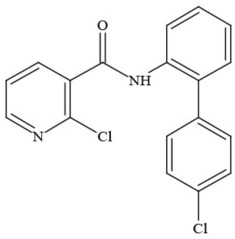	Fungicide	Possible carcinogen; Questionable developmental/reproductive toxin and endocrine disruptor
Carbaryl	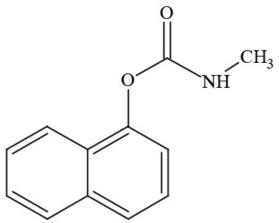	Carbamate/Insecticide	BAD ACTOR; Cholinesterase inhibitor; carcinogen; developmental/reproductive toxin; suspected endocrine disruptor
Carbendazim	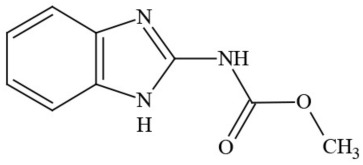	Benzimidalole/Fungicide	Possible carcinogen; questionable ground water contaminant; questionable developmental/reproductive toxin; suspected endocrine disruptor
Clothianidin	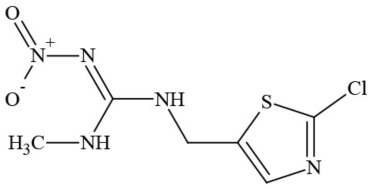	Neonicotinoid/Insecticide	Questionable developmental/reproductive toxin; questionable endocrine disruptor
Diazinon	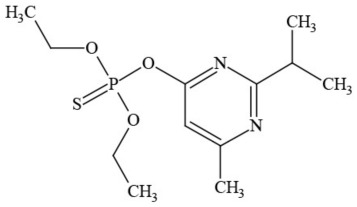	Organophosphate/Insecticide/Miticide	Unlikely carcinogen; cholinesterase inhibitor; developmental/reproductive toxin; suspected endocrine disruptor
Diuron (DCMU)	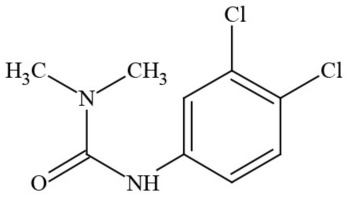	Herbicide/ Photosynthesis inhibitor	Carcinogen; developmental/reproductive toxin; suspected endocrine disruptor
Ethoprophos	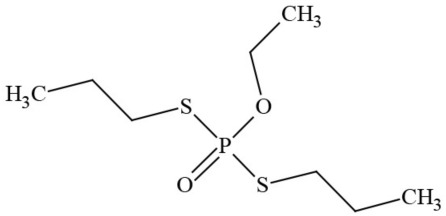	Organophosphate/Insecticide/Nematicide	BAD ACTOR: Carcinogen; cholinesterase inhibitor; potential ground water contaminant; questionable developmental/reproductive toxin; questionable endocrine disruptor
Etoxazole	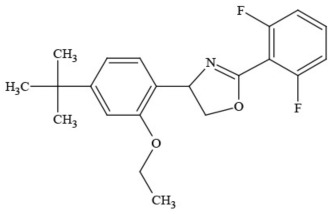	Miticide	Questionable developmental/reproductive toxin and endocrine disruptor
Fenpyroximate	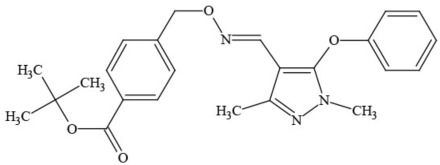	Insecticide/Miticide	Questionable developmental/reproductive toxin; questionable endocrine disruptor
Imidacloprid	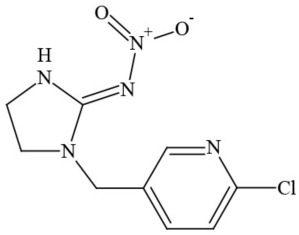	Neonicotinoid/Insecticide	Questionable developmental/reproductive toxin and endocrine disruptor
Malathion	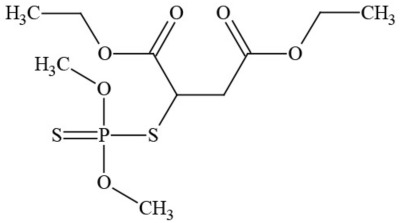	Organophosphate/Insecticide	Cholinesterase inhibitor; possible developmental/reproductive toxin; suspected endocrine disruptor
Myclobutanil	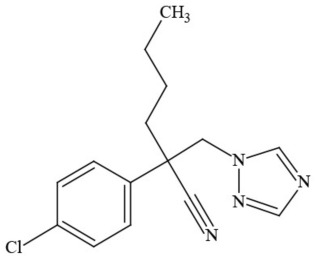	Triazole/Fungicide	Developmental/reproductive toxin; not cholinesterase inhibitor
Permethrin	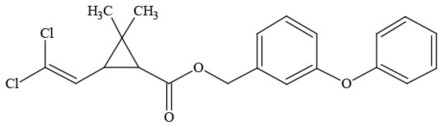	Pyrethroid/Insecticide	BAD ACTOR; Moderate acute toxicity, Carcinogen, potential ground water contaminant, questionable developmental/reproductive toxin, suspected endocrine disruptor
Piperonyl butoxide	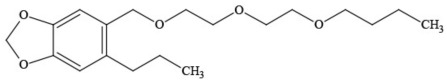	Pesticide synergist	Possible carcinogen; Questionable developmental/reproductive toxin; Suspected endocrine disruptor
Propargite	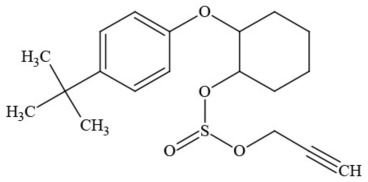	Organochlorine/Miticide	Carcinogen; developmental/reproductive toxin; Questionable endocrine disruptor
Propiconazole	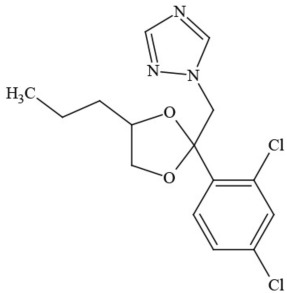	Triazole/Fungicide	Possible carcinogen; developmental/reproductive toxin; Suspected endocrine disruptor
Pyraclostrobin	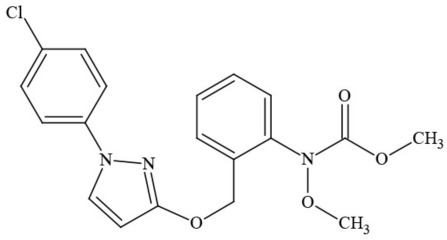	Fungicide	Questionable developmental/reproductive toxin; Questionable endocrine disruptor
Pyriproxyfen	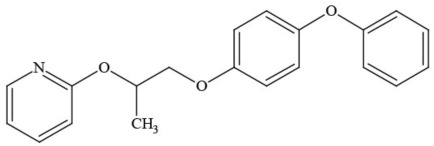	Pyridine/Pesticide	Questionable developmental/reproductive toxin; Questionable endocrine disruptor
Triflumizole	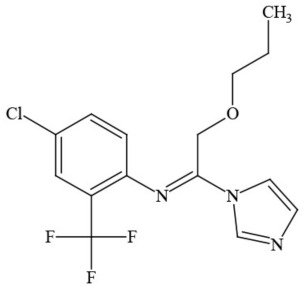	Fungicide	Potential ground water contaminant; Questionable developmental/reproductive toxin; Questionable endocrine disruptor
Trifloxystrobin	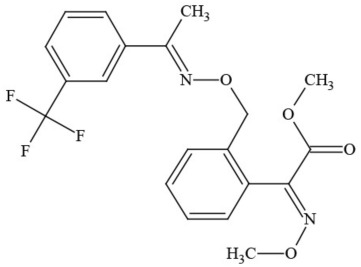	Fungicide	Questionable developmental/reproductive toxin and endocrine disruptor
Triticonazole	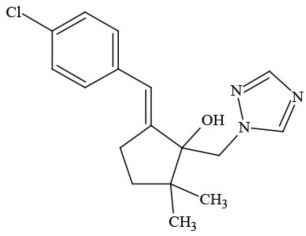	Fungicide	Questionable developmental/reproductive toxin; Questionable endocrine disruptor
Zoxamide	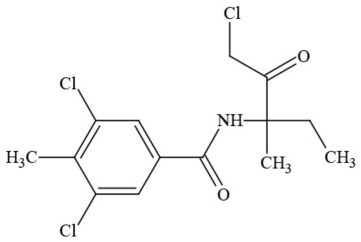	Fungicide	Questionable developmental/reproductive toxin; questionable endocrine disruptor

Data from: American Herbal Pharmacopoeia Cannabis Inflorescence (Loflin and Earleywine, 2014), PAN Pesticides Database: http://www.pesticideinfo.org/Search_Products.jsp#ProdSearch (Fan and Sokorai, 2002), Hemp diseases and pests (Eisenberg et al., 2014). Assay results from Trace Analytics, Spokane, WA. Structures from ACD/ChemSketch 2015.2.5.

The unregulated commerce in cannabis with respect to pesticide usage and lack of available organic certification have resulted in widespread abuse of the legal cannabis market system. Cannabis concentrates currently account for 50% of legal sales in WA, and are also the basis for a burgeoning commerce in cannabis edibles. These products present a clear and present danger, particularly to young patients with epilepsy and other neurological conditions. Future regulation and monitoring with allowance for organic certification and employment of integrated pest management techniques without synthetic pesticides (McPartland et al., 2000) are required approaches to rectify this looming public health threat.

These finding should serve as an indictment of individual industrial oversights and unscrupulous practices, but should not be taken as evidence that cannabis is too dangerous for therapeutic application when proper standards are applied.

## Conclusions

As the legal tide is turning on cannabis as a forbidden drug, experiments are ongoing in the various states (and other countries) as called for in Justice Brandeis' “laboratories of democracy.” Daunting problems remain for those attempting to seek regulatory approval for smoked or vaporized cannabis as a prescription product, whereas nabiximols, a standardized oromucosal spray has achieved such approval in 27 nations based on its ability to demonstrate biochemical consistency, and safety and efficacy in randomized controlled trials. Pure CBD (Epidiolex) also appears headed for regulatory approval.

In contrast, the recreational market is facing numerous challenges in quality control, and addressing myriad safety concerns associated with newer, more potent preparations and novel delivery techniques. Science may provide suitable data for addressing these issues if commensurate research funding is forthcoming to meet the urgent need.

## Author contributions

ER was responsible for the investigation and drafting of this review.

### Conflict of interest statement

The author declares that the research was conducted in the absence of any commercial or financial relationships that could be construed as a potential conflict of interest.

## References

[B1] Abrahamov A. Mechoulam R. (1995). An efficient new cannabinoid antiemetic in pediatric oncology. Life Sci. 56, 2097–2102. 10.1016/0024-3205(95)00194-B 7776837

[B2] Abrams D. I. Hilton J. F. Leiser R. J. Shade S. B. Elbeik T. A. Aweeka F. T.. (2003). Short-term effects of cannabinoids in patients with HIV-1 infection. A randomized, placbo-controlled clinical trial. Ann. Intern. Med. 139, 258–266. 10.7326/0003-4819-139-4-200308190-00008 12965981

[B3] Abrams D. I. Vizoso H. P. Shade S. B. Jay C. Kelly M. E. Benowitz N. L. (2007). Vaporization as a smokeless cannabis delivery system: a pilot study. Clin. Pharmacol. Ther. 82, 572–578. 10.1038/sj.clpt.6100200 17429350

[B4] American Herbal Products Association (2014). Recommendations for Regulators-Cannabis Operations. Silver Spring, MD.

[B5] Bachhuber M. A. Saloner B. Cunningham C. O. Barry C. L. (2014). Medical cannabis laws and opioid analgesic overdose mortality in the United States, 1999-2010. JAMA Intern. Med. 174, 1668–1673. 10.1001/jamainternmed.2014.4005 25154332 PMC4392651

[B6] Backes M. (2014). Cannabis Pharmacy: The Practical Guide to Medical Marijuana. New York, NY: Black Dog & Leventhal.

[B7] Ben-Shabat S. Fride E. Sheskin T. Tamiri T. Rhee M. H. Vogel Z.. (1998). An entourage effect: inactive endogenous fatty acid glycerol esters enhance 2-arachidonoyl-glycerol cannabinoid activity. Eur. J. Pharmacol. 353, 23–31. 10.1016/S0014-2999(98)00392-6 9721036

[B8] Bloor R. N. Wang T. S. Spanel P. Smith D. (2008). Ammonia release from heated ‘street’ cannabis leaf and its potential toxic effects on cannabis users. Addiction 103, 1671–1677. 10.1111/j.1360-0443.2008.02281.x 18705690

[B9] Bornheim L. M. Grillo M. P. (1998). Characterization of cytochrome P450 3A inactivation by cannabidiol: possible involvement of cannabidiol-hydroxyquinone as a P450 inactivator. Chem. Res. Toxicol. 11, 1209–1216. 10.1021/tx9800598 9778318

[B10] Bradford A. C. Bradford W. D. (2016). Medical marijuana laws reduce prescription medication use in medicare part D. Health Aff. (Millwood) 35, 1230–1236. 10.1377/hlthaff.2015.1661 27385238

[B11] Budney A. J. Hughes J. R. Moore B. A. Vandrey R. (2004). Review of the validity and significance of cannabis withdrawal syndrome. Am. J. Psychiatry 161, 1967–1977. 10.1176/appi.ajp.161.11.1967 15514394

[B12] Cabral G. (2001). Immune system, in Cannabis and Cannabinoids: Pharmacology, Toxicology and Therapeutic Potential, eds Grotenhermen F. Russo E. B.(Binghamton, NY: Haworth Press), 279–287.

[B13] Calhoun S. R. Galloway G. P. Smith D. E. (1998). Abuse potential of dronabinol (Marinol). J. Psychoactive Drugs 30, 187–196. 10.1080/02791072.1998.10399689 9692381

[B14] Clarke R. C. (1998). Hashish! Los Angeles, CA: Red Eye Press.

[B15] Collin C. Ehler E. Waberzinek G. Alsindi Z. Davies P. Powell K.. (2010). A double-blind, randomized, placebo-controlled, parallel-group study of Sativex, in subjects with symptoms of spasticity due to multiple sclerosis. Neurol. Res. 32, 451–459. 10.1179/016164109X12590518685660 20307378

[B16] Costa B. Trovato A. E. Comelli F. Giagnoni G. Colleoni M. (2007). The non-psychoactive cannabis constituent cannabidiol is an orally effective therapeutic agent in rat chronic inflammatory and neuropathic pain. Eur. J. Pharmacol. 556, 75–83. 10.1016/j.ejphar.2006.11.006 17157290

[B17] Curran H. V. Freeman T. P. Mokrysz C. Lewis D. A. Morgan C. J. Parsons L. H. (2016). Keep off the grass? Cannabis, cognition and addiction. Nat. Rev. Neurosci. 17, 293–306. 10.1038/nrn.2016.28 27052382

[B18] Degenhardt L. Hall W. Lynskey M. (2003). Testing hypotheses about the relationship between cannabis use and psychosis. Drug Alcohol Depend. 71, 37–48. 10.1016/S0376-8716(03)00064-4 12821204

[B19] Devinsky O. Marsh E. Friedman D. Thiele E. Laux L. Sullivan J.. (2016). Cannabidiol in patients with treatment-resistant epilepsy: an open-label interventional trial. Lancet Neurol. 15, 270–278. 10.1016/S1474-4422(15)00379-8 26724101

[B20] Douthwaite A. H. (1947). Choice of drugs in the treatment of duodenal ulcer. Br. Med. J. 2, 43–47. 10.1136/bmj.2.4514.43 20251793 PMC2055187

[B21] Dworkin R. H. Turk D. C. Farrar J. T. Haythornthwaite J. A. Jensen M. P. Katz N. P.. (2005). Core outcome measures for chronic pain clinical trials: IMMPACT recommendations. Pain 113, 9–19. 10.1016/j.pain.2004.09.012 15621359

[B22] Dyer O. (2016). US states that allow medical marijuana see drop in prescriptions for other drugs, study finds. BMJ 354:i3942. 10.1136/bmj.i3942 27417997

[B23] Earleywine M. Barnwell S. S. (2007). Decreased respiratory symptoms in cannabis users who vaporize. Harm Reduct. J. 4:11. 10.1186/1477-7517-4-11 17437626 PMC1853086

[B24] Eisenberg E. Agintz M. Almog S. (2014). The pharmacokinetics, efficacy, safety, and ease of use of a novel portable metered-dose inhaler in patients with chronic neuropathic pain: A Phase 1a study. J. Pain Palliat. Care Pharmacother. 28, 216–225. 10.3109/15360288.2014.941130 25118789

[B25] Fan X. Gates R. A. (2001). Degradation of monoterpenes in orange juice by gamma radiation. J. Agric. Food Chem. 49, 2422–2426. 10.1021/jf0013813 11368614

[B26] Fan X. Sokorai K. J. (2002). Changes in volatile compounds of gamma-irradiated fresh cilantro leaves during cold storage. J. Agric. Food Chem. 50, 7622–7626. 10.1021/jf020584j 12475280

[B27] Fan X.-H. Cheng Y.-Y. Ye Z.-L. Lin R.-C. and Qian, Z.-Z. (2006). Multiple chromatographic fingerprinting and its application to the quality control of herbal medicines. Anal. Chim. Acta 555, 217–224. 10.1016/j.aca.2005.09.037

[B28] Fischedick J. T. Hazekamp A. Erkelens T. Choi Y. H. Verpoorte R. (2010). Metabolic fingerprinting of *Cannabis sativa* L., cannabinoids and terpenoids for chemotaxonomic and drug standardization purposes. Phytochemistry 71, 2058–2073. 10.1016/j.phytochem.2010.10.001 21040939

[B29] Fitzgerald G. A. (2004). Coxibs and cardiovascular disease. N. Engl. J. Med. 351, 1709–1711. 10.1056/NEJMp048288 15470192

[B30] Food Drug Administration (2004). Guidance for Industry: Botanical Drug Products. U.D.o.H.a.H. Services, US Government.

[B31] Food Drug Administration (2015). Botanical Drug Development Guidance for Industry. U.S.D.o.H.a.H. Services, Food Drug Administration (Washington, DC).

[B32] Foroughi M. Hendson G. Sargent M. A. Steinbok P. (2011). Spontaneous regression of septum pellucidum/forniceal pilocytic astrocytomas–possible role of Cannabis inhalation. Childs. Nerv. Syst. 27, 671–679. 10.1007/s00381-011-1410-4 21336992

[B33] Fride E. (2002). Cannabinoids and cystic fibrosis: a novel approach. J. Cannabis Ther. 2, 59–71. 10.1300/J175v02n01_03

[B34] Fride E. Russo E. B. (2006). Neuropsychiatry: Schizophrenia, depression, and anxiety, in Endocannabinoids: The Brain and Body's Marijuana and Beyond, eds Onaivi E. Sugiura T. Di Marzo V.(Boca Raton, FL: Taylor & Francis), 371–382.

[B35] Gallily R. Yekhtin Z. Hanus L. (2014). Overcoming the bell-shaped dose-response of cannabidiol by using cannabis extract enriched in cannabidiol. Pharmacol. Pharm. 6, 75–85. 10.4236/pp.2015.62010

[B36] Gaoni Y. Mechoulam R. (1964). Isolation, structure and partial synthesis of an active constituent of hashish. J. Am. Chem. Soc. 86, 1646–1647. 10.1021/ja01062a046

[B37] Gertsch J. Leonti M. Raduner S. Racz I. Chen J. Z. Xie X. Q.. (2008). Beta-caryophyllene is a dietary cannabinoid. Proc. Natl. Acad. Sci. U.S.A. 105, 9099–9104. 10.1073/pnas.0803601105 18574142 PMC2449371

[B38] Gieringer D. (1996). Marijuana waterpipe and vaporizer study. MAPS Bull. 6, 59–66.

[B39] Gieringer D. (2001). Cannabis “vaporization": a promising strategy for smoke harm reduction. J. Cannabis Ther. 1, 153–170. 10.1300/J175v01n03_10

[B40] Gieringer D. St. Laurent J. Goodrich S. (2004). Cannabis vaporizer combines efficient delivery of THC with effective suppression of pyrolytic compounds. J. Cannabis Ther. 4, 7–27. 10.1300/J175v04n01_02

[B41] Giese M. W. Lewis M. A. Giese L. Smith K. M. (2015). Development and validation of a reliable and robust method for the analysis of cannabinoids and terpenes in cannabis. J. AOAC Int. 98, 1503–1522. 10.5740/jaoacint.15-116 26651562

[B42] Giri L. Andola H. C. Purohit V. K. Rawat M. S. M. Rawal R. S. Bhatt I. D. (2010). Chromatographic and spectral fingerprinting standardization of traditional medicines: an overview as modern tools. Res. J. Phytochem. 4, 234–241. 10.3923/rjphyto.2010.234.241

[B43] Gottschling S. (2011). Cannbinoide bei Kindern. Gute Erfahrungen bei Schmerzen, Spastik und in der Onkologie. Angewandte Schmerztherapie und Palliativmedizin. 55–57.

[B44] Grinspoon L. Bakalar J. B. (1997). Marihuana, The Forbidden Medicine. New Haven: Yale University Press.

[B45] Grotenhermen F. (2001). Practical hints, in Cannabis and Cannabinoids: Pharmacology, Toxicology and Therapeutic Potential, eds Grotenhermen F. Russo E. B.(Binghamton, NY: Haworth Press), 345–353.

[B46] Grotenhermen F. Leson G. Berghaus G. Drummer O. Krueger H. P. Longo M. (2005). Developing science-based *per se* limits for driving under the influence of cannabis (DUIC), in Findings and Recommendations by an Expert Panel, Nova- Institut, Hürth, Germany, 49.

[B47] Gunn J. K. Rosales C. B. Center K. E. Nuñez A. Gibson S. J. Christ C.. (2016). Prenatal exposure to cannabis and maternal and child health outcomes: a systematic review and meta-analysis. BMJ Open 6:e009986. 10.1136/bmjopen-2015-009986 27048634 PMC4823436

[B48] Guy G. W. Stott C. G. (2005). The development of Sativex- a natural cannabis-based medicine, in Cannabinoids as Therapeutics, ed Mechoulam R. (Basel: Birkhäuser Verlag), 231–263.

[B49] GW Pharmaceuticals (2011). Investigator Brochure Sativex Oromucosal Spray. Salisbury: GW Pharmaceuticals, 170.

[B50] Hadorn D. (2004). A review of cannabis and driving skills, in Medicinal Uses of Cannabis and Cannabinoids, eds Guy G. W. Whittle B. A. Robson P.(London: Pharmaceutical Press), 329–368.

[B51] Hampson A. J. Grimaldi M. Lolic M. Wink D. Rosenthal R. Axelrod J. (2000). Neuroprotective antioxidants from marijuana. Ann. N.Y. Acad. Sci. 899, 274–282. 10.1111/j.1749-6632.2000.tb06193.x 10863546

[B52] Hart C. L. Haney M. Vosburg S. K. Comer S. D. Foltin R. W. (2005). Reinforcing effects of oral Δ^9^-THC in male marijuana smokers in a laboratory choice procedure. Psychopharmacology (Berl.) 181, 237–243. 10.1007/s00213-005-2234-2 15830233

[B53] Hashibe M. Morgenstern H. Cui Y. Tashkin D. P. Zhang Z. F. Cozen W.. (2006). Marijuana use and the risk of lung and upper aerodigestive tract cancers: results of a population-based case-control study. Cancer Epidemiol. Biomarkers Prev. 15, 1829–1834. 10.1158/1055-9965.EPI-06-0330 17035389

[B54] Hazekamp A. (2016). Evaluating the effects of gamma-irradiation for decontamination of medicinal cannabis. Front. Pharmacol. 7:108. 10.3389/fphar.2016.00108 27199751 PMC4847121

[B55] Hazekamp A. Ruhaak R. Zuurman L. van Gerven J. Verpoorte R. (2006). Evaluation of a vaporizing device (Volcano) for the pulmonary administration of tetrahydrocannabinol. J. Pharm. Sci. 95, 1308–1317. 10.1002/jps.20574 16637053

[B56] Hickman M. Vickerman P. Macleod J. Lewis G. Zammit S. Kirkbride J.. (2009). If cannabis caused schizophrenia–how many cannabis users may need to be prevented in order to prevent one case of schizophrenia? England and Wales calculations. Addiction 104, 1856–1861. 10.1111/j.1360-0443.2009.02736.x 19832786

[B57] Hilts P. J. (1994). Is nicotine addictive? It depends on whose criteria you use. New York Times, New York, pp. C3.

[B58] Huestis M. A. (2007). Human cannabinoid pharmacokinetics. Chem. Biodivers. 4, 1770–1804. 10.1002/cbdv.200790152 17712819 PMC2689518

[B59] Ilan A. B. Gevins A. Coleman M. ElSohly M. A. de Wit H. (2005). Neurophysiological and subjective profile of marijuana with varying concentrations of cannabinoids. Behav. Pharmacol. 16, 487–496. 10.1097/00008877-200509000-00023 16148455

[B60] Jensen R. P. Luo W. Pankow J. F. Strongin R. M. Peyton D. H. (2015). Hidden formaldehyde in e-cigarette aerosols. N. Engl. J. Med. 372, 392–394. 10.1056/NEJMc1413069 25607446

[B61] Johnson J. R. Burnell-Nugent M. Lossignol D. Ganae-Motan E. D. Potts R. Fallon M. T. (2010). Multicenter, double-blind, randomized, placebo-controlled, parallel-group study of the efficacy, safety, and tolerability of THC:CBD extract and THC extract in patients with intractable cancer-related pain. J. Pain Symptom Manage. 39, 167–179. 10.1016/j.jpainsymman.2009.06.008 19896326

[B62] Jones R. T. Benowitz N. Bachman J. (1976). Clinical studies of cannabis tolerance and dependence. Ann. N.Y. Acad. Sci. 282, 221–239. 10.1111/j.1749-6632.1976.tb49901.x 798533

[B63] Katona S. Kaminski E. Sanders H. Zajicek J. (2005). Cannabinoid influence on cytokine profile in multiple sclerosis. Clin. Exp. Immunol. 140, 580–585. 10.1111/j.1365-2249.2005.02803.x 15932522 PMC1809378

[B64] Kegley S. E. Hill B. R. Orme S. Choi A. H. (2014). PAN Pesticide Database. Oakland, CA: Pesticide Action Network.

[B65] Koehler J. (2014). Who benefits most from THC:CBD spray? Learning from clinical experience. Eur. Neurol. 71(Suppl. 1), 10–15. 10.1159/000357743 24457847

[B66] Kunos G. (2007). Understanding metabolic homeostasis and imbalance: what is the role of the endocannabinoid system? Am. J. Med. 120, S18–S24; discussion S24. 10.1016/j.amjmed.2007.06.007 17720356

[B67] Lanz C. Mattsson J. Soydaner U. Brenneisen R. (2016). Medicinal cannabis: *in vitro* validation of vaporizers for the smoke-free inhalation of cannabis. PLoS ONE 11:e0147286. 10.1371/journal.pone.0147286 26784441 PMC4718604

[B68] Leweke F. M. Koethe D. Gerth C. W. Nolden B. Mauss C. Schreiber D. (2005). Cannabidiol as an antipsychotic: a double-blind, controlled clinical trial on cannabidiol vs. amisulpride in acute schizophrenia, in Symposium on the Cannabinoids, International Cannabinoid Research Society (Clearwater, FL), 48.

[B69] Leweke F. M. Piomelli D. Pahlisch F. Muhl D. Gerth C. W. Hoyer C.. (2012). Cannabidiol enhances anandamide signaling and alleviates psychotic symptoms of schizophrenia. Transl. Psychiatry 2:e94. 10.1038/tp.2012.15 22832859 PMC3316151

[B70] Loflin M. Earleywine M. (2014). A new method of cannabis ingestion: the dangers of dabs? Addict. Behav. 39, 1430–1433. 10.1016/j.addbeh.2014.05.013 24930049

[B71] Logan B. Kacinko S. L. Beirness D. J. (2016). An Evaluation of Data from Drivers Arrested for Driving Under the Influence in Relation to per se Limits for Cannabis (May 2016). Washington, DC: American Automobile Association Foundation for Traffic Safety.

[B72] Macleod J. Hickman M. (2010). How ideology shapes the evidence and the policy: what do we know about cannabis use and what should we do? Addiction 105, 1326–1330. 10.1111/j.1360-0443.2009.02846.x 20148792

[B73] Macleod J. Davey Smith G. Hickman M. (2006). Does cannabis use cause schizophrenia? Lancet 367, 1055. 10.1016/S0140-6736(06)68468-7 16581402

[B74] Malfait A. M. Gallily R. Sumariwalla P. F. Malik A. S. Andreakos E. Mechoulam R.. (2000). The nonpsychoactive cannabis constituent cannabidiol is an oral anti-arthritic therapeutic in murine collagen-induced arthritis. Proc. Natl. Acad. Sci. U.S.A. 97, 9561–9566. 10.1073/pnas.160105897 10920191 PMC16904

[B75] McPartland J. M. Russo E. B. (2001). Cannabis and cannabis extracts: greater than the sum of their parts? J. Cannabis Ther. 1, 103–132. 10.1300/J175v01n03_08

[B76] McPartland J. M. Russo E. B. (2014). Non-phytocannabinoid constituents of cannabis and herbal synergy, in Handbook of Cannabis, ed Pertwee R. G. (Oxford, UK: Oxford University Press), 280–295.

[B77] McPartland J. M. Clarke R. C. Watson D. P. (2000). Hemp Diseases and Pests: Management and Biological Control. Wallingford, UK: CABI.

[B78] McPartland J. M. Duncan M. Di Marzo V. Pertwee R. G. (2015). Are cannabidiol and Delta(9) -tetrahydrocannabivarin negative modulators of the endocannabinoid system? A systematic review. Br. J. Pharmacol. 172, 737–753. 10.1111/bph.12944 25257544 PMC4301686

[B79] Mechoulam R. Ben-Shabat S. (1999). From gan-zi-gun-nu to anandamide and 2-arachidonoylglycerol: the ongoing story of cannabis. Nat. Prod. Rep. 16, 131–143. 10.1039/a703973e 10331283

[B80] Meier M. H. Hill M. L. Small P. J. Luthar S. S. (2015). Associations of adolescent cannabis use with academic performance and mental health: a longitudinal study of upper middle class youth. Drug Alcohol Depend. 156, 207–212. 10.1016/j.drugalcdep.2015.09.010 26409752 PMC4633365

[B81] Moir D. Rickert W. S. Levasseur G. Larose Y. Maertens R. White P.. (2008). A comparison of mainstream and sidestream marijuana and tobacco cigarette smoke produced under two machine smoking conditions. Chem. Res. Toxicol. 21, 494–502. 10.1021/tx700275p 18062674

[B82] Mokrysz C. Landy R. Gage S. H. Munafò M. R. Roiser J. P. Curran H. V. (2016). Are IQ and educational outcomes in teenagers related to their cannabis use? A prospective cohort study. J. Psychopharmacol. 30, 159–168. 10.1177/0269881115622241 26739345 PMC4724860

[B83] Morgan C. J. Curran H. V. (2008). Effects of cannabidiol on schizophrenia-like symptoms in people who use cannabis. Br. J. Psychiatry 192, 306–307. 10.1192/bjp.bp.107.046649 18378995

[B84] Morgan C. J. Freeman T. P. Schafer G. L. Curran H. V. (2010a). Cannabidiol attenuates the appetitive effects of Delta 9-tetrahydrocannabinol in humans smoking their chosen cannabis. Neuropsychopharmacology 35, 1879–1885. 10.1038/npp.2010.58 20428110 PMC2906701

[B85] Morgan C. J. Schafer G. Freeman T. P. Curran H. V. (2010b). Impact of cannabidiol on the acute memory and psychotomimetic effects of smoked cannabis: naturalistic study. Br. J. Psychiatry 197, 285–290. 10.1192/bjp.bp.110.077503 20884951

[B86] Movig K. L. Mathijssen M. P. Nagel P. H. Van Egmond T. De Gier J. J. Leufkens H. G.. (2004). Psychoactive substance use and the risk of motor vehicle accidents. Accid. Anal. Prev. 36, 631–636. 10.1016/S0001-4575(03)00084-8 15094417

[B87] Müller-Vahl K. R. Emrich H. M. (2008). Cannabis and schizophrenia: towards a cannabinoid hypothesis of schizophrenia. Expert Rev. Neurother. 8, 1037–1048. 10.1586/14737175.8.7.1037 18590475

[B88] Nicholson A. N. Turner C. Stone B. M. Robson P. J. (2004). Effect of delta-9-tetrahydrocannabinol and cannabidiol on nocturnal sleep and early-morning behavior in young adults. J. Clin. Psychopharmacol. 24, 305–313. 10.1097/01.jcp.0000125688.05091.8f 15118485

[B89] Notcutt W. Langford R. Davies P. Ratcliffe S. Potts R. (2012). A placebo-controlled, parallel-group, randomized withdrawal study of subjects with symptoms of spasticity due to multiple sclerosis who are receiving long-term Sativex(R) (nabiximols). Mult. Scler. 18, 219–228. 10.1177/1352458511419700 21878454

[B90] Notcutt W. G. (2013). A questionnaire survey of patients and carers of patients prescribed Sativex as an unlicensed medicine. Prim. Health Care Res. Dev. 14, 192–199. 10.1017/S1463423612000333 22784399

[B91] Novotna A. Mares J. Ratcliffe S. Novakova I. Vachova M. Zapletalova O.. (2011). A randomized, double-blind, placebo-controlled, parallel-group, enriched-design study of nabiximols^*^ (Sativex(®)), as add-on therapy, in subjects with refractory spasticity caused by multiple sclerosis. Eur. J. Neurol. 18, 1122–1131. 10.1111/j.1468-1331.2010.03328.x 21362108

[B92] Nurmikko T. J. Serpell M. G. Hoggart B. Toomey P. J. Morlion B. J. (2005). A multi-center, double-blind, randomized, placebo-controlled trial of oro-mucosal cannabis-based medicine in the treatment of neuropathic pain characterized by allodynia. Neurology 64, A374.

[B93] Nutt D. King L. A. Saulsbury W. Blakemore C. (2007). Development of a rational scale to assess the harm of drugs of potential misuse. Lancet 369, 1047–1053. 10.1016/S0140-6736(07)60464-4 17382831

[B94] O'Keefe K. Earleywine M. (2005). Marijuana Use by Young People: The Impact of State Medical Marijuana Laws. Washington, DC: Marijuana Policy Project, 19.

[B95] Piomelli D. Russo E. B. (2016). The *Cannabis sativa* versus *Cannabis indica* debate: an interview with Ethan Russo, MD. Cannabis Cannabinoid Res. 1, 44–46. 10.1089/can.2015.29003.ebr PMC5576603 28861479

[B96] Pomahacova B. Van der Kooy F. Verpoorte R. (2009). Cannabis smoke condensate III: the cannabinoid content of vaporised *Cannabis sativa*. Inhal. Toxicol. 21, 1108–1112. 10.3109/08958370902748559 19852551

[B97] Pope H. G. Jr. Gruber A. J. Hudson J. I. Huestis M. A. Yurgelun-Todd D. (2001). Neuropsychological performance in long-term cannabis users. Arch. Gen. Psychiatry 58, 909–915. 10.1001/archpsyc.58.10.909 11576028

[B98] Portenoy R. K. Ganae-Motan E. D. Allende S. Yanagihara R. Shaiova L. Weinstein S.. (2012). Nabiximols for opioid-treated cancer patients with poorly-controlled chronic pain: a randomized, placebo-controlled, graded-dose trial. J. Pain 13, 438–449. 10.1016/j.jpain.2012.01.003 22483680

[B99] Potter D. J. (2009). The Propagation, Characterisation and Optimisation of Cannabis sativa L. as a Phytopharmaceutical. Pharmaceutical Sciences, King's College, London, 224.

[B100] Press C. A. Knupp K. G. Chapman K. E. (2015). Parental reporting of response to oral cannabis extracts for treatment of refractory epilepsy. Epilepsy Behav. 45, 49–52. 10.1016/j.yebeh.2015.02.043 25845492

[B101] Raber J. C. Elzinga S. Kaplan C. (2015). Understanding dabs: contamination concerns of cannabis concentrates and cannabinoid transfer during the act of dabbing. J. Toxicol. Sci. 40, 797–803. 10.2131/jts.40.797 26558460

[B102] Rekand T. (2014). THC:CBD spray and MS spasticity symptoms: data from latest studies. Eur. Neurol. 71(Suppl. 1), 4–9. 10.1159/000357742 24457846

[B103] Robson P. (2011). Abuse potential and psychoactive effects of delta-9-tetrahydrocannabinol and cannabidiol oromucosal spray (Sativex), a new cannabinoid medicine. Expert Opin. Drug Saf. 10, 675–685. 10.1517/14740338.2011.575778 21542664

[B104] Rog D. J. Nurmiko T. Friede T. Young C. (2005). Randomized controlled trial of cannabis based medicine in central neuropathic pain due to multiple sclerosis. Neurology 65, 812–819. 10.1212/01.wnl.0000176753.45410.8b 16186518

[B105] Romano L. L. Hazekamp A. (2013). Cannabis oil: chemical evaluation of an upcoming cannabis-based medicine. Cannabinoids 1, 1–11.

[B106] Roques B. (1998). Problèmes Posés Par la Dangerosité des Drogues. Paris: Sécretaire d'Etat à la Santé.

[B107] Russo E. B. (2001). Handbook of Psychotropic Herbs: A Scientific Analysis of Herbal Remedies for Psychiatric Conditions. Binghamton, NY: Haworth Press.

[B108] Russo E. B. (2004). Clinical endocannabinoid deficiency (CECD): can this concept explain therapeutic benefits of cannabis in migraine, fibromyalgia, irritable bowel syndrome and other treatment-resistant conditions? Neuroendocrinol Lett. 25, 31–39. 15159679

[B109] Russo E. B. (2006c). A tale of two cannabinoids: the therapeutic rationale for combining tetrahydrocannabinol (THC) and cannabidiol (CBD). Med. Hypotheses 66, 234–246. 10.1016/j.mehy.2005.08.026 16209908

[B110] Russo E. B. (2006a). The solution to the medicinal cannabis problem, in Ethical Issues in Chronic Pain Management, ed Schatman M. E.(Boca Raton, FL: Taylor & Francis), 165–194.

[B111] Russo E. B. (2006b). The role of cannabis and cannabinoids in pain management, in Weiner's Pain Management: A Practical Guide for Clinicians, eds Cole B. E. Boswell M. (Boca Raton, FL: CRC Press), 823–844.

[B112] Russo E. B. (2008). Cannabinoids in the management of difficult to treat pain. Ther. Clin. Risk Manag. 4, 245–259. 18728714 10.2147/tcrm.s1928 PMC2503660

[B113] Russo E. B. (2011). Taming THC: potential cannabis synergy and phytocannabinoid-terpenoid entourage effects. Br. J. Pharmacol. 163, 1344–1364. 10.1111/j.1476-5381.2011.01238.x 21749363 PMC3165946

[B114] Russo E. B. (2016a). Clinical endocannabinoid deficiency reconsidered: current research supports the theory in migraine, fibromyalgia, irritable bowel, and other treatment-resistant syndromes. Cannabis Cannabinoid Res. 1, 154–165. 10.1089/can.2016.0009 PMC5576607 28861491

[B115] Russo E. B. (2016b). Beyond cannabis: plants and the endocannabinoid system. Trends Pharmacol. Sci. 37, 594–605. 10.1016/j.tips.2016.04.005 27179600

[B116] Russo E. B. (2016c). Pesticide contamination of cannabis in the legal market, 26th Annual Conference on the Cannabinoids, International Cannabinoid Research Society (Bukovina), 66.

[B117] Russo E. B. Hohmann A. G. (2013). Role of cannabinoids in pain management, in Comprehensive Treatment of Chronic Pain by Medical, Interventional and Behavioral Approaches, eds Deer T. Gordin V.(New York, NY: Springer), 181–197.

[B118] Russo E. B. Etges T. Stott C. G. (2008). Comprehensive adverse event profile of Sativex, in 18th Annual Symposium on the Cannabinoids., International Cannabinoid Research Society (Aviemore), 136.

[B119] Russo E. B. Mathre M. L. Byrne A. Velin R. Bach P. J. Sanchez-Ramos J. (2002). Chronic cannabis use in the Compassionate Investigational New Drug Program: an examination of benefits and adverse effects of legal clinical cannabis. J. Cannabis Ther. 2, 3–57. 10.1300/J175v02n01_02

[B120] Russo E. B. Mead A. P. Sulak D. (2015). Current status and future of cannabis research, in Clinical Researcher. 58–63. 10.14524/CR-15-0004

[B121] Samaha A. N. Robinson T. E. (2005). Why does the rapid delivery of drugs to the brain promote addiction? Trends Pharmacol. Sci. 26, 82–87. 10.1016/j.tips.2004.12.007 15681025

[B122] Schoedel K. A. Chen N. Hilliard A. White L. Stott C. Russo E.. (2011). A randomized, double-blind, placebo-controlled, crossover study to evaluate the subjective abuse potential and cognitive effects of nabiximols oromucosal spray in subjects with a history of recreational cannabis use. Hum. Psychopharmacol. 26, 224–236. 10.1002/hup.1196 21671456

[B123] Sellers E. M. Schoedel K. Bartlett C. Romach M. Russo E. B. Stott C. G.. (2013). A multiple-dose, randomized, double-blind, placebo-controlled, parallel-group QT/QTc study to evaluate the electrophysiologic effects of THC/CBD Spray. Clin. Pharmacol. Drug Dev. 2, 285–294. 10.1002/cpdd.36 27121791

[B124] Serpell M. G. Notcutt W. Collin C. (2013). Sativex long-term use: an open-label trial in patients with spasticity due to multiple sclerosis. J. Neurol. 260, 285–295. 10.1007/s00415-012-6634-z 22878432

[B125] Singh R. Sandhu J. Kaur B. Juren T. Steward W. P. Segerbäck D.. (2009). Evaluation of the DNA damaging potential of cannabis cigarette smoke by the determination of acetaldehyde derived N2-ethyl-2′-deoxyguanosine adducts. Chem. Res. Toxicol. 22, 1181–1188. 10.1021/tx900106y 19449825

[B126] Smith N. T. (2002). A review of the published literature into cannabis withdrawal symptoms in human users. Addiction 97, 621–632. 10.1046/j.1360-0443.2002.00026.x 12084124

[B127] Soderstrom C. A. Dischinger P. C. Kufera J. A. Ho S. M. Shepard A. (2005). Crash culpability relative to age, and sex for injured drivers using alcohol, marijuana or cocaine, in Association for the Advancement of Automotive Medicine (Cambridge, MA). PMC3217441 16179157

[B128] Solowij N. Stephens R. S. Roffman R. A. Babor T. Kadden R. Miller M.. (2002). Cognitive functioning of long-term heavy cannabis users seeking treatment. JAMA 287, 1123–1131. 10.1001/jama.287.9.1123 11879109

[B129] Storz M. Russo E. B. (2003). An interview with Markus Storz. J. Cannabis Ther. 3, 67–78. 10.1300/J175v03n01_04

[B130] Stott C. G. Guy G. W. Wright S. Whittle B. A. (2005). The effects of cannabis extracts Tetranabinex & Nabidiolex on human cyclo-oxygenase (COX) activity, in Symposium on the Cannabinoids, International Cannabinoid Research Society (Clearwater, FL).

[B131] Stott C. White L. Wright S. Wilbraham D. Guy G. (2013). A Phase I, open-label, randomized, crossover study in three parallel groups to evaluate the effect of Rifampicin, Ketoconazole, and Omeprazole on the pharmacokinetics of THC/CBD oromucosal spray in healthy volunteers. SpringerPlus 2:236. 10.1186/2193-1801-2-236 23750331 PMC3671111

[B132] Sullivan N. Elzinga S. Raber J. C. (2013). Determination of pesticide residues in cannabis smoke. J. Toxicol. 2013:378168. 10.1155/2013/378168 23737769 PMC3666265

[B133] Tambe Y. Tsujiuchi H. Honda G. Ikeshiro Y. Tanaka S. (1996). Gastric cytoprotection of the non-steroidal anti-inflammatory sesquiterpene, beta-caryophyllene. Planta Med. 62, 469–470. 10.1055/s-2006-957942 9005452

[B134] Tashkin D. P. (2005). Smoked marijuana as a cause of lung injury. Monaldi Arch. Chest Dis. 63, 93–100. 10.4081/monaldi.2005.645 16128224

[B135] Tashkin D. P. (2013). Effects of marijuana smoking on the lung. Ann. Am. Thorac. Soc. 10, 239–247. 10.1513/AnnalsATS.201212-127FR 23802821

[B136] Tashkin D. P. Simmons M. S. Sherrill D. L. Coulson A. H. (1997). Heavy habitual marijuana smoking does not cause an accelerated decline in FEV1 with age. Am. J. Respir. Crit. Care Med. 155, 141–148. 10.1164/ajrccm.155.1.9001303 9001303

[B137] Topol E. J. (2004). Failing the public health–rofecoxib, Merck, and the FDA. N. Engl. J. Med. 351, 1707–1709. 10.1056/NEJMp048286 15470193

[B138] Torres C. A. Hart C. L. (2016). Prenatal Cannabis Exposure and Cognitive Function: A Critical Review. College on Problems of Drug Dependency, Palm Springs, CA, 142.

[B139] Toth C. Au S. (2008). A prospective identification of neuropathic pain in specific chronic polyneuropathy syndromes and response to pharmacological therapy. Pain 138, 657–666. 10.1016/j.pain.2008.04.023 18691815

[B140] Tuttle A. H. Tohyama S. Ramsay T. Kimmelman J. Schweinhardt P. Bennett G. J.. (2015). Increasing placebo responses over time in U.S. clinical trials of neuropathic pain. Pain 156, 2616–2626. 10.1097/j.pain.0000000000000333 26307858

[B141] Tyler V. E. (1993). Phytomedicines in Western Europe: potential impact on herbal medicine in the United States, in Human Medicinal Agents from Plants, ACS Symposium, No. 534, eds Kinghorn A. D. Balandrin M. F.(Philadelphia: American Chemical Society), 25–37.

[B142] United States Commission on Marihuana Drug Abuse (1972). Marihuana: A Signal of Misunderstanding. First Report, U.S. Government Publishing Office, Washington.

[B143] Upton R. Craker L. ElSohly M. Romm A. Russo E. Sexton M. (2013). Cannabis Inflorescence: Cannabis spp.: Standards of Identity, Analysis and Quality Control. Scotts Valley, CA: American Herbal Pharmacopoeia.

[B144] U.S. General Accounting Office (2002). Marijuana: Early Experiences with Four States' Laws that Allow Use For Medical Purposes. Washington, DC: United States General Accounting Office, 63.

[B145] Van der Kooy F. Pomahacova B. Verpoorte R. (2008). Cannabis smoke condensate I: the effect of different preparation methods on tetrahydrocannabinol levels. Inhal. Toxicol. 20, 801–804. 10.1080/08958370802013559 18645719

[B146] Vandrey R. Raber J. C. Raber M. E. Douglass B. Miller C. Bonn-Miller M. O. (2015). Cannabinoid dose and label accuracy in edible medical cannabis products. JAMA 313, 2491–2493. 10.1001/jama.2015.6613 26103034

[B147] Verster J. C. Volkerts E. R. (2004). Antihistamines and driving ability: evidence from on-the-road driving studies during normal traffic. Ann. Allergy Asthma Immunol. 92, 294–303; quiz 303–355. 10.1016/S1081-1206(10)61566-9 15049392

[B148] Wachtel S. R. ElSohly M. A. Ross R. A. Ambre J. de Wit H. (2002). Comparison of the subjective effects of delta9-tetrahydrocannabinol and marijuana in humans. Psychopharmacology 161, 331–339. 10.1007/s00213-002-1033-2 12073159

[B149] Wade D. (2012). Evaluation of the safety and tolerability profile of Sativex: is it reassuring enough? Expert Rev. Neurother. 12, 9–14. 10.1586/ern.12.12 22509986

[B150] Wade D. T. Collin C. Stott C. Duncombe P. (2010). Meta-analysis of the efficacy and safety of Sativex (nabiximols), on spasticity in people with multiple sclerosis. Mult. Scler. 16, 707–714. 10.1177/1352458510367462 20558502

[B151] Wade D. T. Makela P. Robson P. House H. Bateman C. (2004). Do cannabis-based medicinal extracts have general or specific effects on symptoms in multiple sclerosis? A double-blind, randomized, placebo-controlled study on 160 patients. Mult. Scler. 10, 434–441. 10.1191/1352458504ms1082oa 15327042

[B152] Wade D. T. Makela P. M. House H. Bateman C. Robson P. J. (2006). Long-term use of a cannabis-based medicine in the treatment of spasticity and other symptoms in multiple sclerosis. Multiple Scler. 12, 639–645. 10.1177/1352458505070618 17086911

[B153] Walsh Z. Gonzales R. Crosby K. Carroll C. Bonn-Miller M. O. (2016). Medical cannabis and mental health: a systematic review. Clin. Psychol. Rev. 27816801 10.1016/j.cpr.2016.10.002

[B154] Ware M. Wang W. Shapiro S. Ducruet T. Robinson A. Gamsa A. (2007). Smoked cannabis for chronic neuropathic pain: results of a pilot study, in 17th Annual Symposium on the Cannabinoids (Saint-Sauveur, QC: International Cannabinoid Research Society), 31.

[B155] Whittle B. A. Guy G. W. Robson P. (2001). Prospects for new cannabis-based prescription medicines. J. Cannabis Ther. 1, 183–205. 10.1300/J175v01n03_12

[B156] Wilkinson J. D. Whalley B. J. Baker D. Pryce G. Constanti A. Gibbons S.. (2003). Medicinal cannabis: is delta9-tetrahydrocannabinol necessary for all its effects? J. Pharm. Pharmacol. 55, 1687–1694. 10.1211/0022357022304 14738597

[B157] Williamson E. M. (2001). Synergy and other interactions in phytomedicines. Phytomedicine 8, 401–409. 10.1078/0944-7113-00060 11695885

[B158] Wright S. Duncombe P. Altman D. G. (2012). Assessment of blinding to treatment allocation in studies of a cannabis-based medicine (Sativex(R)) in people with multiple sclerosis: a new approach. Trials 13:189. 10.1186/1745-6215-13-189 23046749 PMC3487910

[B159] Yücel M. Lorenzetti V. Suo C. Zalesky A. Fornito A. Takagi M. J.. (2016). Hippocampal harms, protection and recovery following regular cannabis use. Transl. Psychiatry 6, e710. 10.1038/tp.2015.201 26756903 PMC5068875

[B160] Zajicek J. Fox P. Sanders H. Wright D. Vickery J. Nunn A.. (2003). Cannabinoids for treatment of spasticity and other symptoms related to multiple sclerosis (CAMS study): multicentre randomised placebo-controlled trial. Lancet 362, 1517–1526. 10.1016/S0140-6736(03)14738-1 14615106

[B161] Zuardi A. W. Guimaraes F. S. (1997). Cannabidiol as an anxiolytic and antipsychotic, in Cannabis in Medical Practice: A Legal, Historical and Pharmacological Overview of the Therapeutic Use of Marijuana, ed Mathre M. L.(Jefferson, NC: McFarland), 133–141.

[B162] Zuardi A. W. Morais S. L. Guimarães F. S. Mechoulam R. (1995). Antipsychotic effect of cannabidiol [letter]. J. Clin. Psychiatry 56, 485–486. 7559378

[B163] Zuardi A. W. Shirakawa I. Finkelfarb E. Karniol I. G. (1982). Action of cannabidiol on the anxiety and other effects produced by delta 9-THC in normal subjects. Psychopharmacology 76, 245–250. 10.1007/BF00432554 6285406

[B164] Zuurman L. Roy C. Schoemaker R. C. Hazekamp A. den Hartigh J. Bender J. C.. (2008). Effect of intrapulmonary tetrahydrocannabinol administration in humans. J. Psychopharmacol. 22, 707–716. 10.1177/0269881108089581 18515447

